# Global phylogeny of *Treponema pallidum* lineages reveals recent expansion and spread of contemporary syphilis

**DOI:** 10.1038/s41564-021-01000-z

**Published:** 2021-11-24

**Authors:** Mathew A. Beale, Michael Marks, Michelle J. Cole, Min-Kuang Lee, Rachel Pitt, Christopher Ruis, Eszter Balla, Tania Crucitti, Michael Ewens, Candela Fernández-Naval, Anna Grankvist, Malcolm Guiver, Chris R. Kenyon, Rafil Khairullin, Ranmini Kularatne, Maider Arando, Barbara J. Molini, Andrey Obukhov, Emma E. Page, Fruzsina Petrovay, Cornelis Rietmeijer, Dominic Rowley, Sandy Shokoples, Erasmus Smit, Emma L. Sweeney, George Taiaroa, Jaime H. Vera, Christine Wennerås, David M. Whiley, Deborah A. Williamson, Gwenda Hughes, Prenilla Naidu, Magnus Unemo, Mel Krajden, Sheila A. Lukehart, Muhammad G. Morshed, Helen Fifer, Nicholas R. Thomson

**Affiliations:** 1 Parasites and Microbes Programme, Wellcome Sanger Institute, Hinxton, UK; 2 Faculty of Infectious and Tropical Diseases, London School of Hygiene and Tropical Medicine, London, UK; 3 Hospital for Tropical Diseases, University College London Hospitals NHS Foundation Trust, London, UK; 4 HCAI, Fungal, AMR, AMU and Sepsis Division, UK Health Security Agency, London, UK; 5 British Columbia Centre for Disease Control, Public Health Laboratory, Vancouver, British Columbia Canada; 6 Molecular Immunity Unit, MRC-Laboratory of Molecular Biology, Department of Medicine, University of Cambridge, Cambridge, UK; 7 Department of Veterinary Medicine, University of Cambridge, Cambridge, UK; 8 Bacterial STIs Reference Laboratory, Department of Bacteriology, National Public Health Centre, Budapest, Hungary; 9 Department of Clinical Sciences, Institute of Tropical Medicine, Antwerpen, Belgium; 10 Brotherton Wing Clinic, Brotherton Wing, Leeds General Infirmary, Leeds, UK; 11 Microbiology Department, Vall d’Hebron Research Institute, Universitat Autònoma de Barcelona, Barcelona, Spain; 12 National Reference Laboratory for STIs, Department of Clinical Microbiology, Sahlgrenska University Hospital, Gothenburg, Sweden; 13 Laboratory Network, Manchester, UK Health Security Agency, Manchester Royal Infirmary, Manchester, UK; 14 Institute of Fundamental Medicine and Biology, Kazan Federal University, Kazan, Russia; 15 Centre for HIV and STI, National Institute for Communicable Diseases, Johannesburg, South Africa; 16 STI Unit Vall d’Hebron-Drassanes, Infectious Diseases Department, Hospital Vall d’Hebron, Barcelona, Spain; 17 Department of Medicine, University of Washington, Seattle, WA USA; 18 Tuvan Republican Skin and Venereal Diseases Dispensary, Ministry of Health of Tuva Republic, Kyzyl, Russia; 19 Virology Department, Old Medical School, Leeds Teaching Hospitals Trust, Leeds, UK; 20 Colorado School of Public Health, University of Colorado, Denver, CO USA; 21 Midlands Regional Hospital Portlaoise, Laois, Ireland; 22 Alberta Precision Laboratories, Edmonton, Alberta Canada; 23 Clinical Microbiology Department, Queen Elizabeth Hospital, Birmingham, UK; 24 Institute of Environmental Science and Research, Wellington, New Zealand; 25 The University of Queensland Centre for Clinical Research, Faculty of Medicine, The University of Queensland, Brisbane, Queensland Australia; 26 Department of Microbiology and Immunology, The Peter Doherty Institute for Infection and Immunity, Melbourne, Victoria Australia; 27 Department of Global Health and Infection, Brighton and Sussex Medical School, University of Sussex, Brighton, UK; 28 Department of Infectious Diseases, Institute of Biomedicine, University of Gothenburg, Gothenburg, Sweden; 29 Pathology Queensland Central Laboratory, Brisbane, Queensland Australia; 30 Department of Infectious Disease Epidemiology, London School of Hygiene and Tropical Medicine, London, UK; 31 Department of Laboratory Medicine and Pathology, Faculty of Medicine, University of Alberta, Edmonton, Alberta Canada; 32 WHO Collaborating Centre for Gonorrhoea and other Sexually Transmitted Infections, National Reference Laboratory for STIs, Faculty of Medicine and Health, Örebro University, Örebro, Sweden; 33 Department of Pathology and Laboratory Medicine, University of British Columbia, Vancouver, British Columbia Canada; 34 Departments of Medicine/Infectious Diseases and Global Health, University of Washington, Seattle, WA USA; 35 Blood Safety, Hepatitis, STI and HIV Division, UK Health Security Agency, London, UK

**Keywords:** Bacterial genomics, Genetic variation, Phylogeny, Bacterial infection, Clinical microbiology

## Abstract

Syphilis, which is caused by the sexually transmitted bacterium *Treponema pallidum* subsp. *pallidum*, has an estimated 6.3 million cases worldwide per annum. In the past ten years, the incidence of syphilis has increased by more than 150% in some high-income countries, but the evolution and epidemiology of the epidemic are poorly understood. To characterize the global population structure of *T. pallidum*, we assembled a geographically and temporally diverse collection of 726 genomes from 626 clinical and 100 laboratory samples collected in 23 countries. We applied phylogenetic analyses and clustering, and found that the global syphilis population comprises just two deeply branching lineages, Nichols and SS14. Both lineages are currently circulating in 12 of the 23 countries sampled. We subdivided *T. p.* *pallidum* into 17 distinct sublineages to provide further phylodynamic resolution. Importantly, two Nichols sublineages have expanded clonally across 9 countries contemporaneously with SS14. Moreover, pairwise genome analyses revealed examples of isolates collected within the last 20 years from 14 different countries that had genetically identical core genomes, which might indicate frequent exchange through international transmission. It is striking that most samples collected before 1983 are phylogenetically distinct from more recently isolated sublineages. Using Bayesian temporal analysis, we detected a population bottleneck occurring during the late 1990s, followed by rapid population expansion in the 2000s that was driven by the dominant *T. pallidum* sublineages circulating today. This expansion may be linked to changing epidemiology, immune evasion or fitness under antimicrobial selection pressure, since many of the contemporary syphilis lineages we have characterized are resistant to macrolides.

## Abstract (web summary)

Global syphilis prevalence has been increasing. Sequencing and analysis of a global collection of 726 *Treponema pallidum* samples reveal globally circulating lineages linked to a rapid expansion occurring since the end of the twentieth century.

## Main

Syphilis, caused by the bacterium *Treponema pallidum* subsp. *pallidum* (TPA), is a prevalent sexually transmitted infection that can cause severe long-term sequelae when left untreated. Historically, syphilis is commonly believed to have caused a large epidemic across Renaissance Europe, having previously been absent or unrecognized^1,2^. Although the origins of syphilis and the accurate dating of the most recent common ancestor of TPA are still the subject of debate^3–6^, it is suggested that the strains of TPA that persist in human populations today can be traced back to its introduction into Western Europe approximately 500 years ago, and its subsequent dissemination globally^3,4,6^.

Following the introduction of effective antibiotics after World War II, syphilis incidence fluctuated^7^ without disappearing, until the 1980s and 1990s during the HIV/AIDS crisis when disease incidence declined markedly^8^, linked to community-wide changes in sexual behaviour, shifting of affected populations, AIDS-related mortality and widespread antimicrobial prophylaxis of HIV-infected populations. However, since the beginning of the twenty-first century, there has been a substantial resurgence in syphilis incidence^9–14^. In many countries, this has been associated with populations of men who have sex with men (MSM) engaging in high-risk sexual activity^11,15^. Transmission between MSM and heterosexuals is a particular concern due to the risk of in utero transmission to the fetus, leading to congenital syphilis^16^.

Previous genomic analyses of TPA genomes have described two deep-branching phylogenetic lineages, ‘SS14’ and ‘Nichols’^3^. SS14-lineage strains represent the vast majority of published genomes^4^, and phylogenetic analysis showed that the origins of the SS14 lineage can be traced back to the 1950s^3^, followed by subsequent expansion of sublineages occurring during the 1990s^4^. Our understanding of the Nichols lineage is predominantly limited to laboratory strains from the USA, with relatively few clinical strains sequenced^4,17^. However, most TPA genomes published to date originate from the USA^4^, Western Europe^3,4,17,18^ and China^19,20^, and our understanding of the true breadth of diversity of TPA is incomplete^21^. Our view of the diversity of syphilis samples pre-dating the twenty-first century is even more limited, and these issues are partly explained by the fact that it has not been possible to culture TPA outside of a rabbit until recently^22^.

In this multi-centre collaborative study, we used direct whole genome sequencing (WGS) to generate a global view of contemporary syphilis from patients in Africa, Asia, the Caribbean, South America and Australia sampled between 1951 and 2019. Our dataset also includes a detailed analysis of the ‘within-country’ variation seen in TPA genomes in North America and Europe. We present evidence of globally spanning transmission networks with identical strains found in dispersed countries, indicating that, based on our data, TPA is genetically homogenous. Furthermore, we show that this genetic homogeneity is the result of a rapid and global expansion of TPA sublineages occurring within the last 30 years following a population bottleneck. This means that the TPA population infecting patients in the twenty-first century is not the same as that infecting patients in the twentieth century.

## Describing the global population structure of *T. p.pallidum*

We performed targeted sequence-capture whole genome sequencing on residual genomic DNA extracted from diagnostic swabs taken from TPA PCR-positive syphilis patients and on TPA strains previously isolated in rabbits. We combined these data with 133 previously published genomes^3,4,18–20,23–26^. After assessment for genome coverage and quality, we had a total of 726 genomes with >25% of genome positions at >5X coverage (mean 82%, range 25–97%), which is sufficient for primary lineage classification. This dataset included 577 new genomes sequenced directly from clinical samples and 16 new genomes sequenced from samples passaged in rabbits.

**Fig. 1:**
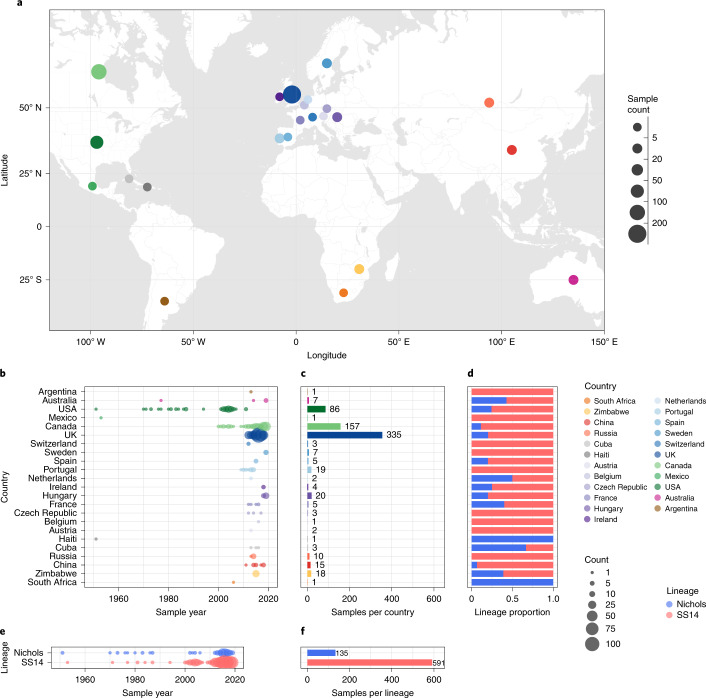
Global distribution of 726 *T. p.pallidum* partial genomes. **a**, Map of sampled countries for 726 partial (>25% of genome positions) genomes. Circle size corresponds to total number of genomes (binned into categories), and colour corresponds to country. Country coordinates used are the country centroid position, except for Russia (where the centroid for the Tuva Republic is used) and Mexico (where the location of Mexico City is used). Map tiles by Stamen Design (CC-BY 3.0), map data by OpenStreetMap (ODbl). **b**, Temporal distribution of samples by country. Size of circle indicates number of samples for that year. Three samples from Baltimore (USA) had uncertain sampling dates (1960–1980) and were set to 1970 for plotting dates in **b** and **e**. Genomes derived from passaged variants of the Nichols-1912 isolate or those with uncertain collection dates are not shown in plotted timeline (**b**, **e**). **c**, Total count of samples by country. **d**, Relative proportion of country samples corresponding to each TPA lineage (where only one sample was present per country, this shows the lineage it corresponds to). **e**, Temporal distribution of the samples by TPA lineage. **f**, Total count of samples by TPA lineage.

Our dataset includes 23 countries (range 1–355 genomes per country; Fig. 1a,c), including previously poorly or unsampled regions such as Africa (South Africa (*n* = 1) and Zimbabwe (*n* = 18)), Scandinavia (Sweden, *n* = 7), Central Europe (Hungary, *n* = 20), Central Asia (Tuva Republic, Russia, *n* = 10) and Australia (*n* = 5), as well as substantially increasing the sampling from North America (Canada, *n* = 157; the USA, *n* = 86) and Western Europe (Spain, *n* = 5; Belgium, *n* = 1; Ireland, *n* = 4; the UK, *n* = 355). We also included previously published genomes from South America (Argentina, *n* = 1), the Caribbean (Cuba, *n* = 3; Haiti, *n* = 1) and elsewhere. Due to a lack of long-term archived samples, 96.0% (*n* = 697) of samples were collected from 2000 onwards (Fig. 1b,e). Samples collected before 2000 (*n* = 29) were passaged in the in vivo rabbit model (Supplementary Data 1), whereas most samples collected from 2000 onwards (89.8%, 626/697) were sequenced directly from clinical samples.

**Extended Data Fig. 1:**
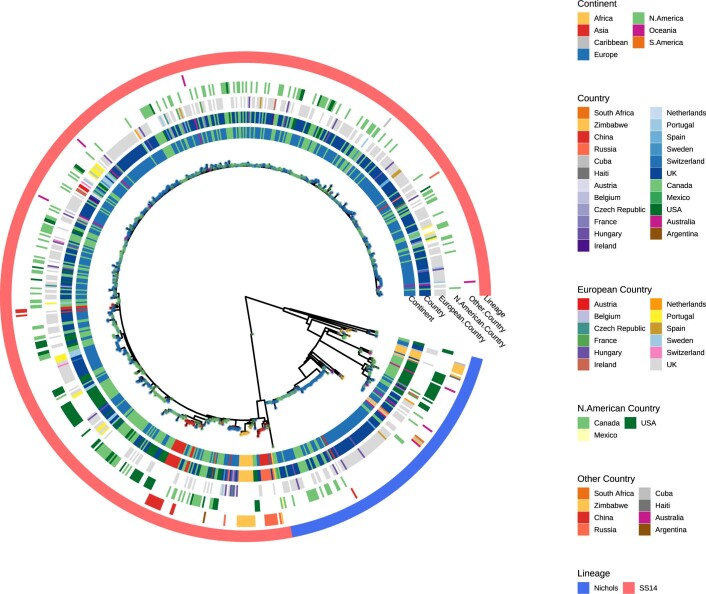
Phylogenetic distribution of 726 *Treponema pallidum* ssp *pallidum* partial genomes. Maximum likelihood phylogeny of 726 partial (>25% of genome positions) genomes shows two primary lineages (Nichols, SS14), with no obvious correlation by country or continent. Tree tip points are coloured by continent. Coloured strips show continent, country (all), countries separated by region (European Countries, North American Countries, Other Countries) and TPA lineage. One very low coverage sample (TPA_BCC144, Canada, 47% genome breadth, 7.9X mean coverage) appears basal to the SS14-lineage clade in this phylogeny, but due to low coverage it was not possible to determine the correct placement.

Phylogenetic analysis assigned all genomes into one of two deeply branching lineages (Nichols or SS14) (Extended Data Fig. 1). Looking across all well-sampled countries (Fig. 1c,d), from the first detection of the modern SS14 lineage (excluding the outlying 1953 Mexico A strain) in the 1970s, we consistently see both lineages circulating broadly through until 2019 (Fig. 1b,e). More specifically, 81.3% (*n* = 590) of the genomes belonged to the SS14 lineage and 18.7% (*n* = 136) belonged to the Nichols lineage, and in the 12 out of 23 countries where both lineages were present, 80.3% (544/677, median per country 75.3%, range 33.3–93.3%) were of the SS14 lineage (Fig. 1d,f). Of the 11 countries showing only a single lineage, most had three or fewer samples, the notable exceptions being Portugal (*n* = 19), Sweden (*n* = 7) and Russia (*n* = 10) (Fig. 1c).

## Fine-scale analysis of SS14- and Nichols-lineage phylogenies

**Fig. 2:**
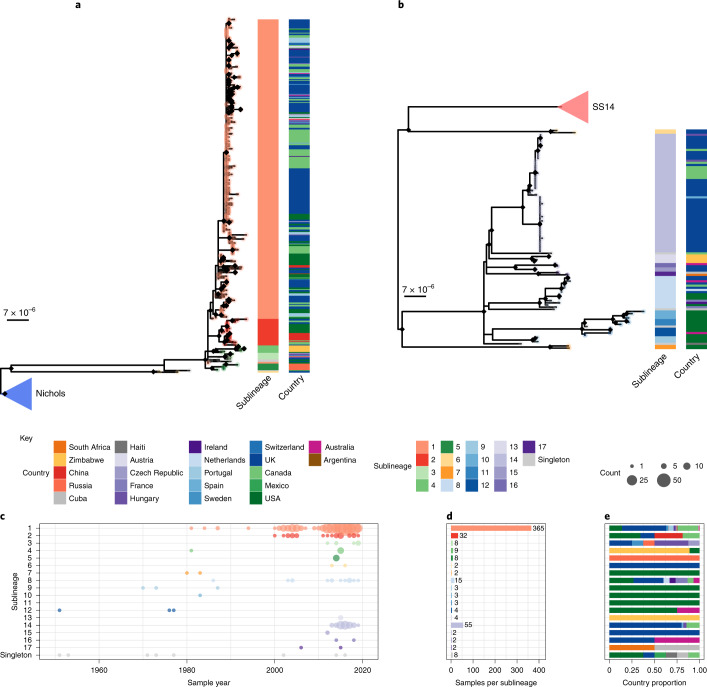
Fine-scale analysis of 528 high-quality (>75% reference sites) TPA genomes and sublineages. **a**, Recombination-masked WGS phylogeny, highlighting the SS14 lineage (*n* = 426). **b**, Recombination-masked WGS phylogeny, highlighting the Nichols lineage (*n* = 102), including four outlying genomes (sublineages 6 and 7). For **a** and **b**, coloured strips show sublineage and country; tree tips show sublineage. Coloured triangle indicates node position of collapsed sister lineage. UltraFast bootstraps ≥95% are indicated with black diamond node marks. Note that sublineage 6 is shown in both trees (see main text). **c**, Temporal distribution of samples by sublineage (unrelated singleton genomes are grouped together). **d**, Total count of samples by sublineage. **e**, Relative proportion of each sublineage sample corresponding to country.

**Extended Data Fig. 2:**
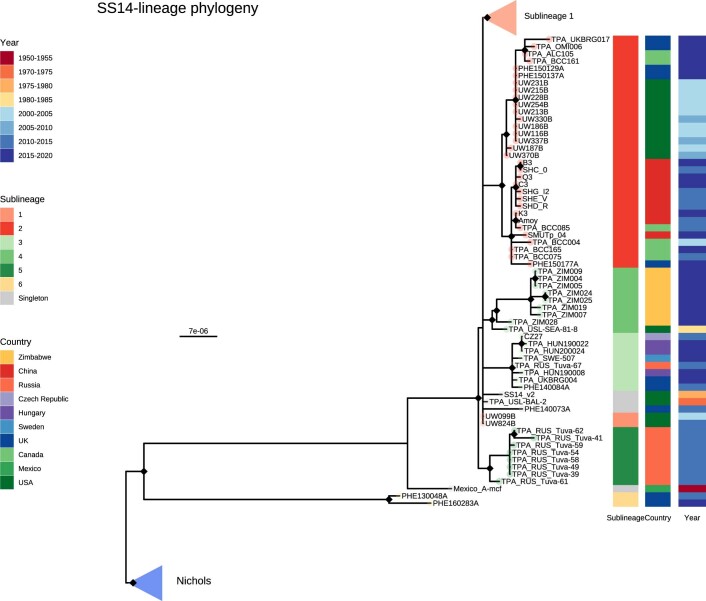
Detailed subtree of SS14-lineage. Recombination masked WGS phylogeny, showing the SS14-lineage and sublineages. The low diversity globally distributed sublineage 1 has been collapsed to enable visualization of the remaining sublineages. Tip points are coloured by sublineage, and coloured strips show sublineage and country. Blue triangle indicates collapsed Nichols-lineage, pink triangle indicates collapsed sublineage 1. Two samples close to the root of the common SS14-lineage clades were clustered as sublineage 1, and are shown. Note that sublineage 6 diverges from a node close to the root of TPA, and appears on the SS14 side in this midpoint rooted tree.

**Extended Data Fig. 3:**
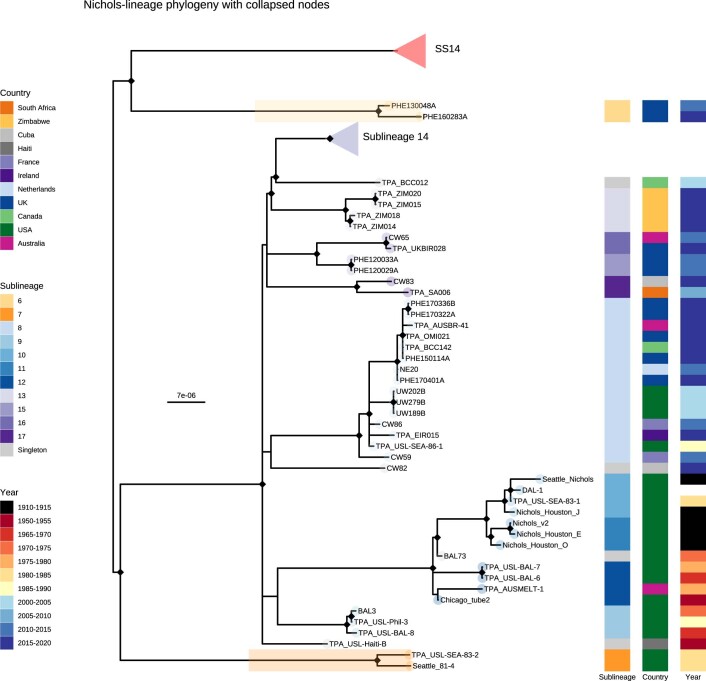
Subtree highlighting novel Nichols-lineage strains. Recombination masked WGS phylogeny, showing the Nichols-lineage and sublineages. Tip points are coloured by sublineage, and coloured strips show sublineage and country. Shaded boxes highlight basal Nichols-lineage outgroup sublineages 6 and 7. Note that sublineage 6 diverges from a node close to the root of TPA, and appears on the SS14 side in this midpoint rooted tree. The large clonal sublineage 14 has been collapsed to enable clearer visualization of the remaining taxa. The pink triangle indicates collapsed SS14-lineage, blue triangle indicates the collapsed sublineage 14.

**Extended Data Fig. 4:**
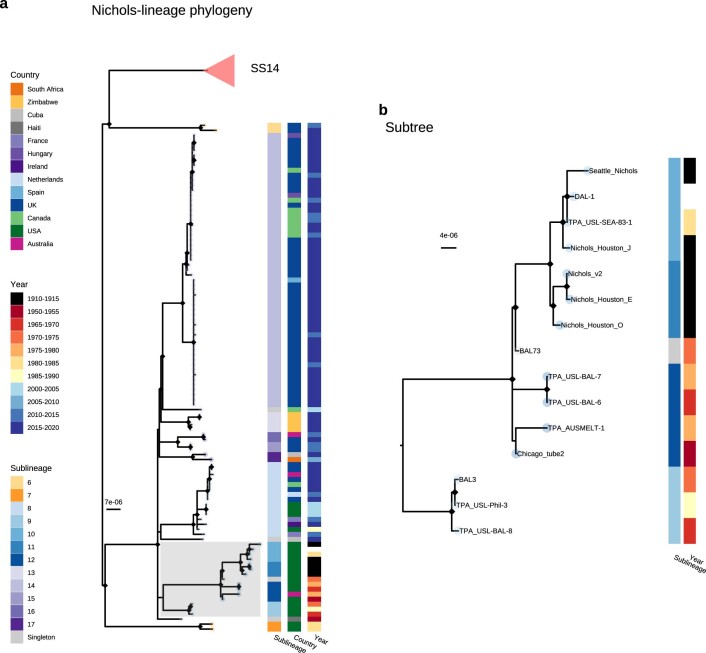
Commonly used Nichols Reference genomes form a monophyletic clade distinct from contemporary clinical strains. A- Recombination masked WGS phylogeny, showing the Nichols-lineage and sublineages. Shaded grey box shows a monophyletic clade containing commonly used reference genomes as well as genetically related strains. Tip points are coloured by sublineage, and coloured strips show sublineage and country. Pink triangle indicates collapsed SS14-lineage. B- Expanded view of a seemingly extinct clade containing common reference strains including Nichols_v2, DAL-1 and Seattle_Nichols. The most recent sample closely related to the reference strains (TPA_USL-SEA-83-1) was collected in 1983, while the latest sample for the entire clade (TPA_USL-Phil-3) was collected in 1987. The provenance of the sample originally used for sequencing the DAL-1 genome is uncertain, but in the literature the original isolation was in 1988. The placement of both DAL-1 and TPA_USL-SEA-83-1 within the diversity of Nichols-1912 derivatives suggests the possibility of the samples being mislabeled in the handling laboratories.

To answer finer-scale evolutionary questions, we filtered our dataset to focus on the 528 genomes with >75% genome sites at >5X (genome length 1,139,569 bp, mean % sites 92.9%, range 75.1–96.9%) and a mean coverage of 111X (range 11X–727X). This filtered dataset comprised 401 new and 127 published genomes (Supplementary Data 1), but excluded the only sample from Belgium, leaving 22 countries in the analysis. After excluding 19 regions of recombination and genomic uncertainty due to gene paralogy or repetitive regions^3,4,6^, we used Gubbins^27^ to infer a further 19 regions of putative recombination (see Methods and Supplementary Data 2 for details). We refer to the resulting masked sequence alignment as the core genome and used it to infer a whole genome maximum likelihood phylogeny using IQ-Tree^28^ (Supplementary Fig. 1). To define sublineage clusters, we used 100 bootstrapped trees as independent inputs to rPinecone^29^ with a 10 single nucleotide polymorphism (SNP) threshold as previously described^4^, and evaluated their consistency using hierarchical clustering (Supplementary Fig. 2). We found broad support for the Nichols sublineages across all conditions evaluated, but some parts of the SS14-lineage phylogeny were less well supported. To focus on the more stable sublineages, we required that at least 5% of the bootstrap replicates supported a cluster (the most conservative threshold tested – see Supplementary Fig. 2 and Methods). Using this approach, we defined 17 sublineages and 8 singleton strains across both SS14 and Nichols lineages (SS14: 426 genomes divided into 5 sublineages and 4 singletons; Nichols: 102 genomes divided into 12 sublineages and 4 singletons; Fig. 2, Supplementary Fig. 1 and Extended Data Figs. 2–4). Sublineage 6 diverged from other genomes very close to the common ancestor of TPA, and due to low total diversity in the dataset can appear on either side of the root (Nichols or SS14) depending on the phylogenetic approach used; for the purposes of this analysis, we classified sublineage 6 strains as Nichols lineage since they are more distantly related to the recent contemporary SS14-lineage expansions.

From Fig. 2, it is apparent that the phylogeny of the SS14 lineage is dominated by SS14 sublineage 1 (*n* = 365), composed of closely related genomes present in 18 countries and 6 continental groupings (Asia, Caribbean, Europe, North America, Oceania and South America). The oldest example of sublineage 1 was collected in 1981 (TPA_USL-SEA-81-3, Seattle, USA) and the most recent samples were from 2019 (Fig. 2c). Sublineage 2 (*n* = 32; Fig. 2e and Extended Data Fig. 2) contained samples from Canada, China, the UK and the USA. In a previous analysis^4^, we manually divided this sublineage into two groups (one from China, one from the USA) on the basis of temporal and geospatial divergence, and the independent evolution of different macrolide resistance alleles. However, by adding new genomes (Extended Data Fig. 2), we now see that even the original cluster of samples from China is interspersed with genomes from the UK (*n* = 1) and Canada (*n* = 4), indicating that this is not a geographically restricted group.

The twelve 2015 Zimbabwean genomes in our study formed two distinct clades, one nested within the SS14 lineage (sublineage 4, *n* = 8, also including a distantly related singleton from the US 1981 sublineage, TPA_USL-SEA-81-8) and the other within the Nichols lineage (sublineage 13, *n* = 4) and exclusively found in Zimbabwe. We also examined 10 genomes collected in the Tuva Republic, central Russia in 2013/2014, and these were distributed between three different SS14 sublineages (1, 3, 5). Sublineage 5 was found only in Tuva, while sublineage 3 was found throughout Europe (Czech Republic, Hungary, Sweden and the UK; Fig. 2e and Extended Data Fig. 2), with the remaining sample from Russia belonging to the highly expanded global SS14 sublineage 1.

Consistent with previous studies^3,4,17^, Nichols-lineage strains were genetically more diverse, with longer branch lengths and higher nucleotide diversity than SS14-lineage strains (Nichols π = 3.2 × 10^−5^, SS14 π = 6 × 10^−6^), reflecting the predicted time of lineage diversification. However, our increased sampling also revealed two recent clonal expansions within the Nichols lineage: sublineage 14 (*n* = 55), comprising samples from Canada, Hungary, Spain and the UK (Fig. 2e) and sublineage 8 (*n* = 15), comprising samples from Australia, Canada, France, Ireland, the Netherlands, the UK and the USA (Extended Data Fig. 3).

In addition to observing evidence of recent clonal expansions, we also show greater resolution of Nichols-lineage diversity, identifying two new samples from UK patients (PHE130048A and PHE160283A collected in 2013 and 2016, respectively), which occupy positions basal to all Nichols-lineage strains (Extended Data Fig. 3). Indeed, this analysis suggests that the most recent common ancestor of this sublineage was very close to the root of all TPA.

**Extended Data Fig. 5:**
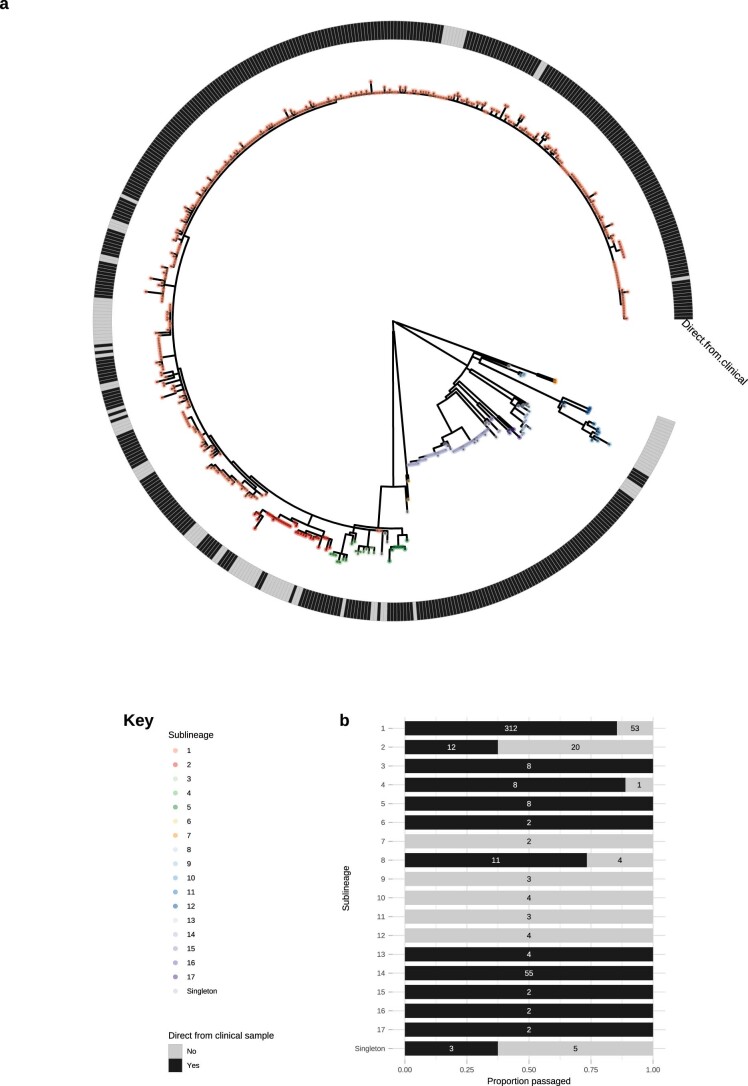
Finescale analysis of 528 high quality TPA genomes and sublineages, showing distribution of samples sequenced directly from clinical samples and those passaged in rabbit model. A – Whole genome phylogeny showing distribution of samples sequenced directly from clinical sample or rabbit-passaged. B – Distribution of samples sequenced directly from clinical sample and rabbit-passaged samples according to sublineage, showing proportion (bar) and exact count (number). For both A and B, plots are coloured according to being directly sequenced from clinical samples (Black) or after rabbit passage (grey). Samples passaged in rabbits are distributed throughout the global TPA phylogeny, and present in 9/17 sublineages. Older samples from before 2000 were isolated via rabbit passage, and dominate extinct clusters, as well as clustering close to the most recent common ancestor of contemporary sublineages such as SS14 sublineage 1.

Multiple derivatives of the highly passaged prototype Nichols-lineage strain (denoted Nichols-1912) isolated in 1912 were included in our analysis (Nichols_v2, Seattle_Nichols, Nichols_Houston_E, Nichols_Houston_J and Nichols_Houston_O). Figure 2b (Extended Data Fig. 4) shows that derivatives of Nichols-1912 fall within a distinct clade that also includes independently collected contemporaneous clinical samples (some with minimal passage through the rabbit model), including a sample (TPA_AUSMELT-1) collected in Australia in 1977^30^. This clade could be subdivided into four sublineages (9, 10, 11, 12) and one singleton. Notably, the last sample belonging to this clade was collected in 1987 (TPA_USL-Phil-3). Hence, within our sampling framework, this appears to be an example of a historic lineage declining to rarity or even becoming extinct. More broadly, we note that although this cluster of both clinical and laboratory strains were all passaged to varying degrees through the rabbit model, other samples passaged in the rabbit model were distributed throughout the phylogeny and were present in 9 out of 17 sublineages (Extended Data Fig. 5).

### Temporal analysis of population dispersal

To infer temporal patterns within the global phylogeny, we performed Bayesian phylogenetic reconstruction using BEAST^31^ under a Strict Clock model with a Bayesian Skyline population distribution. We excluded 8 samples from strains known to be heavily passaged or with uncertain collection dates from the previous dataset of 528, giving a dataset of 520 samples and 883 variable sites. We inferred a median molecular clock rate of 1.28 × 10^−7^ substitutions per site per year (95% highest posterior density 1.07 × 10^−7^–1.48 × 10^−7^), which is equivalent to one substitution per genome every 6.9 years, consistent with recent analyses^4,6^.

**Fig. 3:**
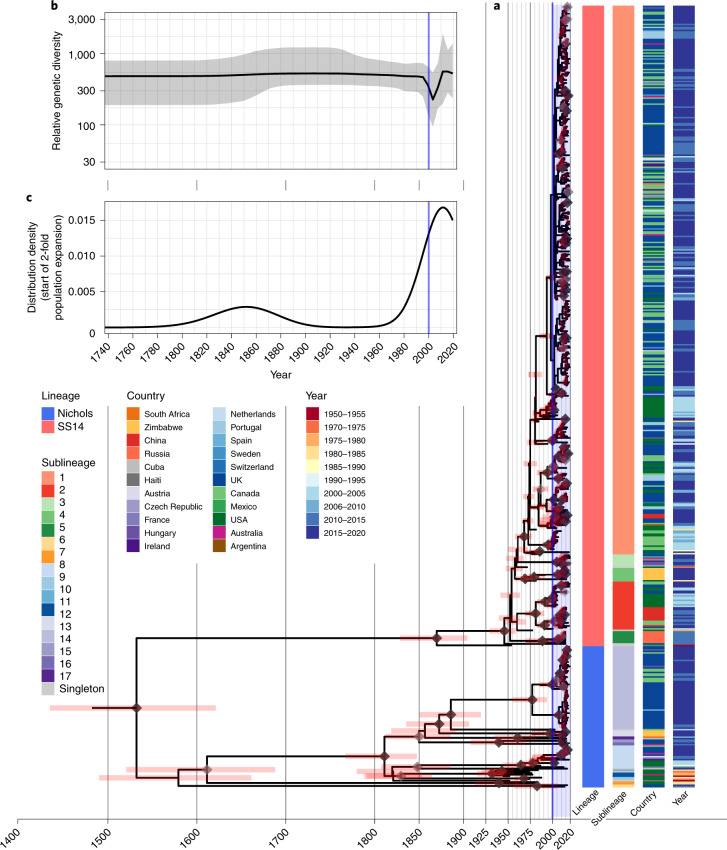
Bayesian maximum-credibility phylogeny of 520 TPA genomes shows population contraction during the 1990s, followed by rapid expansion from the early 2000s onwards. **a**, Time-scaled phylogeny of 520 TPA genomes. Node points are shaded according to posterior support (black ≥96%, dark grey >91%, light grey >80%). Pink bars on nodes indicate 95% highest posterior density intervals. Blue line and shaded area highlight post-2000 expansion of lineages. **b**, Bayesian Skyline plot of genetic diversity shows small population expansion and contractions during the nineteenth and twentieth centuries, followed by a sharp decline and rapid re-emergence during the 1990s and 2000s. **c**, Posterior distribution of start dates for a 2-fold expansion above Skyline mean shows strong support for expansion after 1990 in 68.6% (9,263/13,503) of trees.

Within the global TPA phylogeny (Fig. 3a), we observed several patterns of genomic dispersal. The first reflects the separation of the Nichols and SS14 lineages (median date in our analysis 1534, 95% highest posterior density 1430–1621), which is the subject of much previous analysis^3,4,6^. These data also showed that the common ancestor of these lineages was separated from recent samples by long phylogenetic branches and an absence of older ancestral nodes, suggesting unsampled historical diversity and that most contemporary TPA descended from much more recent ancestral nodes. We previously dated the common ancestors for clonal expansions of 9 SS14 sublineages between the 1980s and early 2000s^4^. With this expanded dataset, we focused on the major clonally expanded sublineages 1, 2, 8 and 14, each having at least 15 samples.

**Extended Data Fig. 6:**
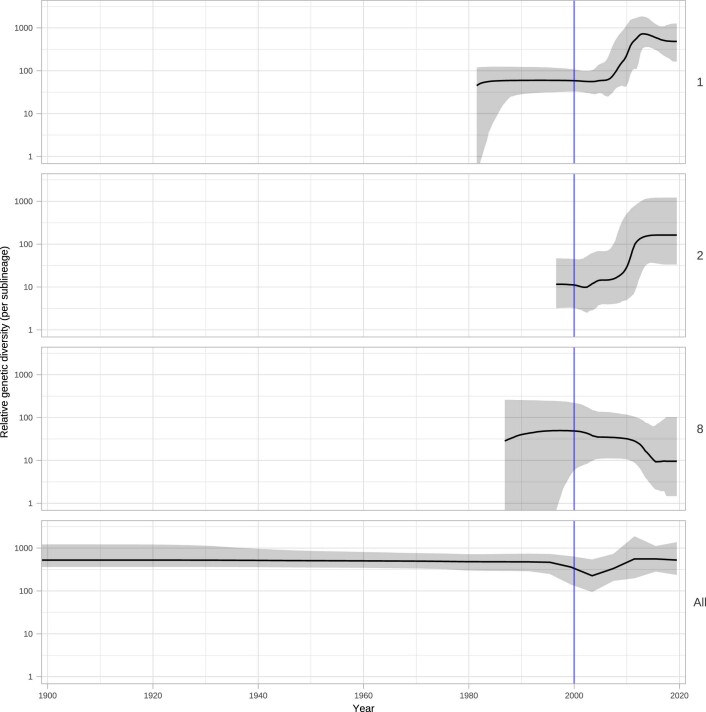
Bayesian Skyline analysis of sublineages. Plots show population expansions occurring during the early 2000s for all sublineages with >15 samples apart from sublineage 14. Sublineage 14, which had low temporal signal, did not converge after multiple BEAST runs. Shows Skyline plots of sublineages 1, 2, 8 and plot for all samples from Fig. 3.

**Extended Data Fig. 7:**
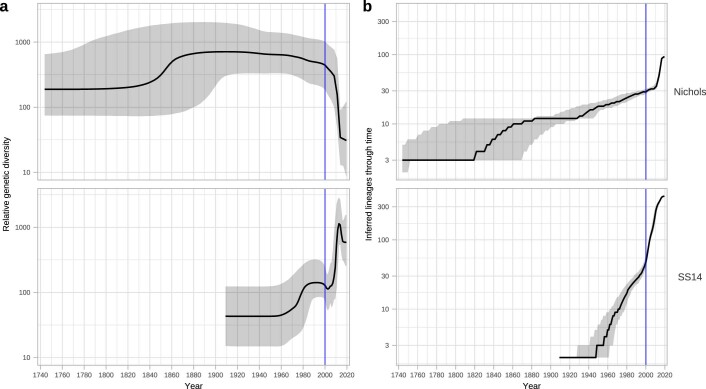
Independent analysis of SS14 and Nichols phylodynamics shows differential patterns of expansion and decline. A – Independent Bayesian Skyline plots for Nichols- and SS14- lineages. B – Independent lineages through time plot for Nichols- and SS14-lineages. Skyline analysis indicates expansion of SS14-lineage after 2000 coincided with a decline of Nichols-lineage. However, analysis of lineage accumulation through time shows that both SS14 and Nichols continued to expand after 2000; whilst this is visible as a steep slope from 2000 in SS14, rapid expansion for Nichols occurred after 2010.

Next, we used Bayesian Skyline analysis to determine the relative genetic diversity over different time periods in the phylogeny (Fig. 3b), and found a very sharp decline during the 1990s and 2000s, followed by an equally sharp rise that continued until the present. To test the statistical support for this expansion, we extracted the proportion of trees in the posterior distribution supporting a >2-fold population expansion above the population mean (68.6%, 9,263/13,503 trees) and plotted the distribution of expansion start dates (Fig. 3c) (median date 2011). We further tested the proportion of trees supporting a 2-fold population decline between 1990–2015 (90.7%, 12,248/13,503 trees, median date 2000) and a 2-fold population expansion between 1990–2015 (59.0%, 7,966/13,503 trees, median date 2012) (Supplementary Fig. 3). These findings were also apparent in SS14 sublineages 1 and 2 (Extended Data Fig. 6), but not in Nichols sublineage 8. We had insufficient temporal signal to repeat this analysis on multi-country expanded Nichols sublineage 14 (Supplementary Fig. 4). We independently analysed the SS14 and Nichols lineages, and results indicated that the population decline was largely associated with the Nichols lineage and coincided with expansion of the SS14 lineage (Extended Data Fig. 7a). However, our analysis also shows that the Nichols lineage continued to diversify after 2010 (Extended Data Fig. 7b), consistent with our analysis of clonally expanded Nichols sublineages.

### Global sharing of sublineages and identical strains

**Fig. 4:**
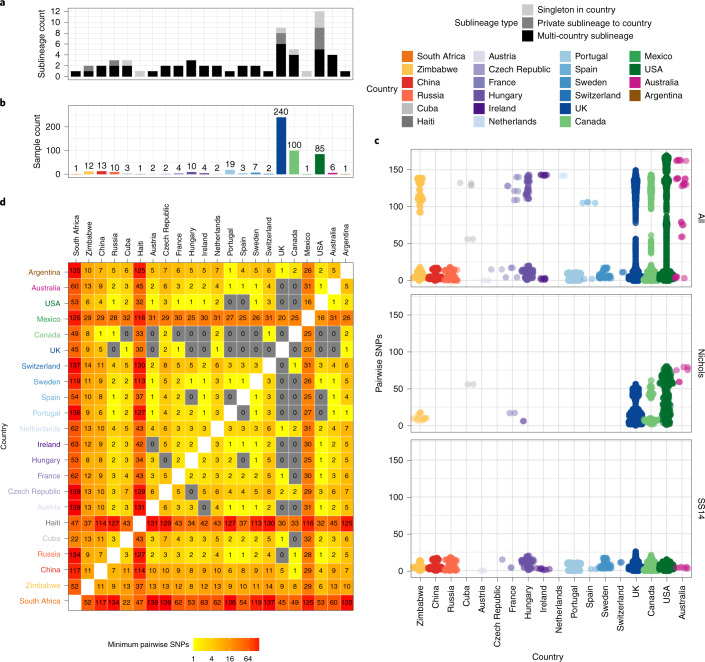
Substantial sharing of closely related strains within and between countries. **a**, Number of sublineages found per country, classified by sublineage distribution (multi-country, black; private to one country, medium grey; singleton, light grey). **b**, Total high-quality genomes per country. **c**, Pairwise comparison of SNP distance distributions from samples in each country (with >1 sample), across all samples and within lineages. **d**, Minimum pairwise SNPs between samples from different countries. All pairwise SNP comparisons exclude comparisons with same samples. Haiti, South Africa and Mexico appear to be striking outliers in terms of genetic relatedness, but this reflects that the Haiti and Mexico samples were collected in the 1950s, and we had only a single genome from these countries.

**Extended Data Fig. 8:**
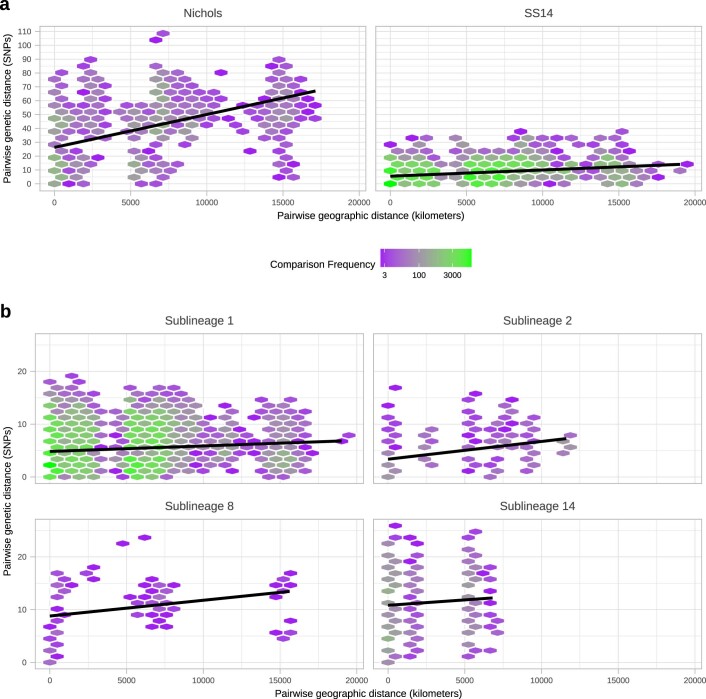
Effect of geographic distance on genetic distance. A- Pairwise comparison of genetic distance (SNPs) and geographic distance (km; calculated using country centroids) within Nichols- and SS14-lineages, including linear regression (95% CI not visible). B- Pairwise comparison of genetic distance (SNPs) and geographic distance (km; calculated using country centroids) within the four major multi-country sublineages (SS14: 1, 2; Nichols: 8, 14). Includes linear regression (95% CI not visible).

To further understand the patterns of recent population expansion, we sought evidence of sharing of sublineages among countries, classifying sublineages as singletons (*n* = 8), private to a country (*n* = 8) or multi-country (*n* = 9), and found that 20 out of 22 countries contained at least one multi-country sublineage (Fig. 4a and Supplementary Fig. 5). We inferred pairwise SNP distances for genomes within and between each country (Fig. 4c,d), and where there was more than one sample (*n* = 18), we found fewer than 26 (SS14 lineage) and 80 (Nichols lineage) pairwise SNPs separating genomes within any one country (Fig. 4c), illustrating the close genetic relationships between samples (particularly for the SS14 lineage). We also found very low genetic distance between paired samples from different countries, with 27 country pairings (14 countries) showing zero core genome pairwise SNPs (Fig. 4d). In particular, Canada, the UK and the USA with the highest sampling (Fig. 4b), showed the most zero pairwise interactions with other countries (Fig. 4d). Therefore, we cannot rule out similar transmission events occurring between other countries. We compared pairwise SNP distances with geographical distance between country centroids. Although we found a moderate correlation for Nichols lineage (Pearson’s correlation 0.49, *P* < 0.001, 9,620 comparisons), correlation was lower for the SS14 lineage (0.31, *P* < 0.001, 181,476 comparisons) and for the four largest multi-country sublineages (sublineage 1: 0.09, *P* < 0.001, 133,225 comparisons; sublineage 2: 0.43, *P* < 0.001, 1,024 comparisons; sublineage 8: 0.27, *P* < 0.001, 225 comparisons; sublineage 14: 0.08, *P* < 0.001, 3,025 comparisons) (Extended Data Fig. 8). Hence, overall this indicates weak geographical structure for TPA.

**Extended Data Fig. 9:**
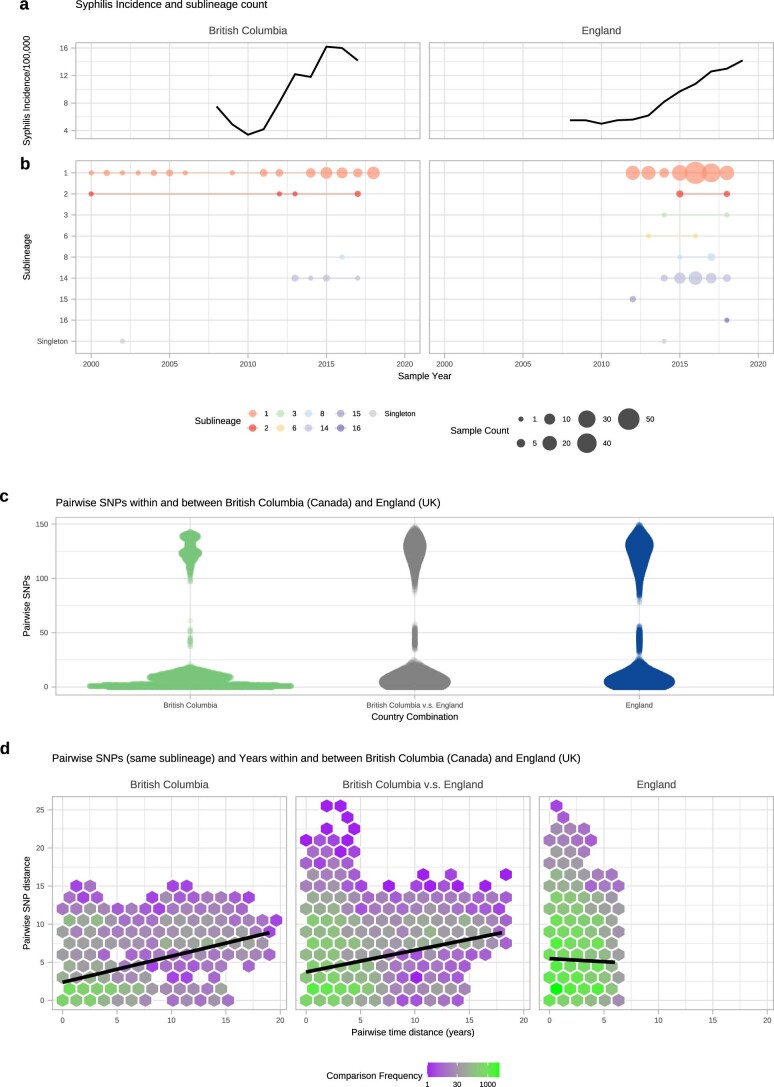
Sharing of sublineages and closely related strains within and between British Columbia (Canada) and England (UK). A- Syphilis incidence per 100,000 population by year for British Columbia, (Canada) and England (UK) using currently published data. B- TPA sublineage counts for each year, using high quality genomes from British Columbia (n = 84) and England (n = 240). British Columbia samples collected from 2000-2018, English samples collected from 2012-2018. C- Pairwise comparison of SNP distance distributions from samples within and between British Columbia and England. D- Comparison of SNP distance and temporal distance within and between British Columbia and England. The plot is divided into hexagonal bins, with the colour of each hexagon representing the number of comparisons (white = none, purple = few, green = many, see scale). Linear regression lines also shown (95% CI not visible).

To understand these observations more fully, we focused on British Columbia (Canada) and England, both of which have experienced a recent rise in syphilis incidence (Extended Data Fig. 9a), and for which we had a large number of samples. Included were 84 high-quality BC genomes collected by the BC Centre for Disease Control between 2000 and 2018. From England, we had 240 high quality genomes from samples collected by the National Reference Laboratory at Public Health England (*n* = 198) and four non-referring laboratories (*n* = 42) between 2012 and 2018. In BC, SS14 sublineage 1 dominated throughout the 18 year survey period, representing 82% of all BC genomes (Extended Data Fig. 9b). In addition, isolated cases of SS14 sublineage 2 were seen in 2000 and 2012, as well as a single Nichols-lineage sample (singleton) in 2002 (Extended Data Fig. 9b). Then from 2013 onwards, we detected two new Nichols sublineages: Nichols sublineage 8 and sublineage 14. The latter two lineages were also found across the USA and Europe (Fig. 2e).

Both the Nichols and the SS14 lineages were consistently present in the English samples between 2012 and 2018. All of the common sublineages (4/4) found in BC were also present in England, as well as 4 additional sublineages (Nichols sublineages 6, 15, 16; SS14 sublineage 3) and one SS14 singleton strain not detected in BC (Extended Data Fig. 9b). Sublineage 14, which was first detected in BC in 2013, was also the most numerous Nichols sublineage in England, but was not detected here until 2014.

Pairwise SNP distances between orthogonal genomes from the same sublineage showed 2,622 pairwise combinations of BC (*n* = 56) and English samples (*n* = 78) sharing zero pairwise SNPs over the core genome alignment for isolates collected between 2004 and 2019. To understand the effect of temporal distance, we compared both the pairwise SNP distance and the pairwise time distance (in years) between genomes from the same sublineage (Extended Data Fig. 9d). These data showed that the mean number of years separating identical core genomes was 2.5 years (range 0–15), and the mean temporal distances of identical genomes were similar within BC (2.9 years), within England (1.9 years) and between the two (2.7 years). The number of pairwise SNPs increased with temporal separation across all BC and English genomes from the same sublineage (Pearson’s correlation 0.126, *P* < 0.001, 55,841 comparisons), with a mean of 4.9 SNPs (range 0–23) separating genomes from the same year and sublineage, compared with 7.8 SNPs (range 6–11) after 19 years (Extended Data Fig. 9d). This means that inference of direct patient-to-patient TPA transmission using the core genome will be challenging at the population level, and limits opportunities for real-time genomic epidemiology because identical genomes can be separated by many years, hence confidence intervals around temporal reconstructions will be broad. In the case of sublineage 14, we first detected this in BC, then the following year in England. Since we had a deeply sampled survey of populations over time for both countries, it seems plausible that this represents a novel introduction into BC and England. However, low temporal rates and incomplete sampling mean that this must be interpreted with caution.

We also found some rarer sublineages – either as singleton strains or those private to a single country. While this might be expected in poorly sampled and geographically distant locations, such as Cuba, Haiti, Mexico and Zimbabwe, we found that the majority of private (6/8) and singleton (5/8) sublineages were actually from Canada, the UK or the USA (Fig. 4a), suggesting that deeper sampling elsewhere may also find novel diversity.

**Extended Data Fig. 10:**
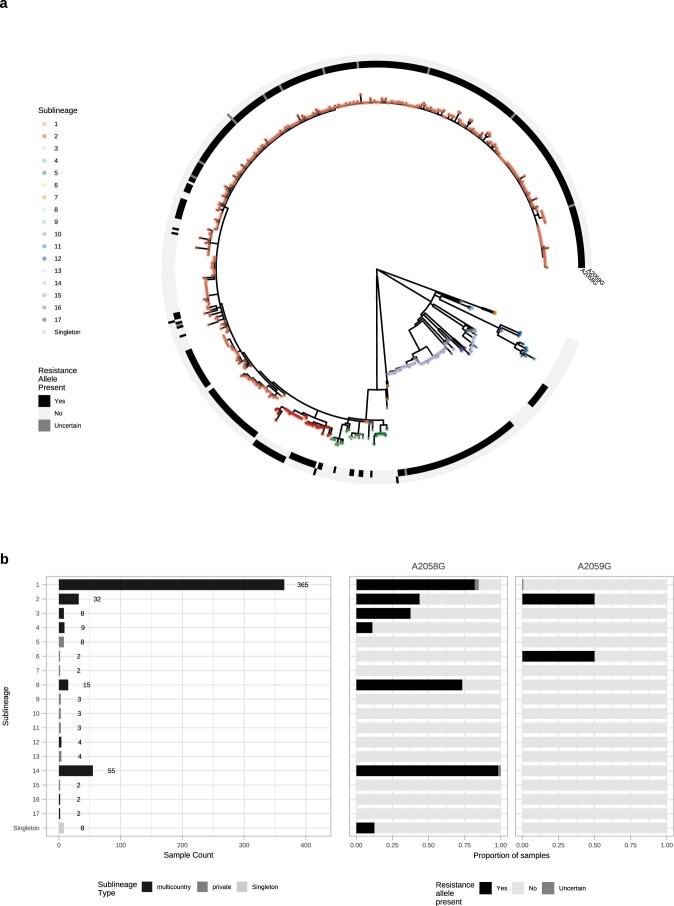
Multicountry sublineages are broadly macrolide resistant. A- Whole genome phylogeny showing distribution of macrolide resistance conferring SNPs (A2058G and A2059G). B- Distribution of macrolide resistance SNPs by sublineage, indicating number of samples per sublineage, and sublineage type. Note that while the common A2058G mutation was found in six sublineages (both Nichols- and SS14-lineages), we also found the less common A2059G in both SS14-lineage (sublineages 1, 2) and Nichols-lineage (sublineage 6).

Given our observations of individual sublineage expansion, we investigated whether the expansion could be related to antimicrobial resistance. Overlaying SNPs known to confer macrolide resistance (A2058G, A2059G) in the ribosomal 23S rRNA gene on the phylogeny showed evidence of macrolide resistance in 6 out of 9 multi-country sublineage expansions (Extended Data Fig. 10), with the majority of samples being resistant in the largest sublineages 1, 2, 8 and 14. In contrast, only one private sublineage (sublineage 6, *n* = 2) contained a macrolide-resistant sample, suggesting that macrolide resistance is potentially linked to expansion in multi-country sublineages.

We further explored the genomic differences between SS14 and Nichols lineages in our core genome alignment, using ancestral reconstruction to infer the common ancestral sequences of contemporary SS14 and Nichols lineages (Supplementary Fig. 6). We identified 95 SNPs separating these clades, and functional annotation indicated that 16 SNPs in 11 genes resulted in non-synonymous coding changes, 59 SNPs were synonymous and 20 were intragenic (Supplementary Data 3). We noted a high number of non-synonymous changes to the *bamA* gene (TPASS_RS01600; involved in outer-membrane protein assembly), but none of these genes is known to confer a fitness advantage that may distinguish the two lineages.

## Discussion

Previous attempts to understand the origins of the original syphilis pandemic^3,6,21^, as well as the dynamics of the current one^3,4^, have been constrained by the technical difficulty in sequencing TPA genomes, as well as relatively small datasets with limited geographical diversity and sampling biases. In our study, we assembled the most comprehensive collection of syphilis samples from around the world to date, including samples from both the twentieth and twenty-first centuries. Despite this, we still find that the TPA population consists of just two deep-branching lineages, the SS14 and the Nichols lineage, with no outlying lineages. We showed that these lineages are both globally distributed and, where we have densely sampled, we found the relative proportions of each to be consistent. Although the overall diversity detected within the Nichols lineage is far greater than that of the SS14 lineage, suggesting earlier dissemination, we also found that these two major lineages exhibit similar phylodynamics, with recent sublineage expansions being apparent in both lineages. This suggests that both the Nichols and the SS14 lineages are capable of exploiting the transmission pathways driving the current syphilis epidemic.

Among our data, we sequenced the first genomes from syphilis patients in Africa, and our analysis shows that these genomes represented novel private sublineages, but their genomic diversity is nested entirely within the existing phylogenetic framework – these TPA genomes are not unusual. Indeed, we even observed the same pattern of Nichols and SS14 lineages, both being present in Zimbabwe, suggesting multiple introduction events into Zimbabwe. The same was true for genomes sequenced from Central Russia, where the private sublineage 5 represented novel, but entirely unremarkable genomic diversity.

In our study, we found that sublineages and genetically similar genomes were more likely to be shared among deeply sampled countries. This suggests that sublineage sharing between countries is high and deeper sampling of other countries will probably reveal similar patterns of sharing. As sampling depth increased, we also found rarer sublineages, notably sublineage 6, representing novel outlying genetic diversity basal to all contemporary Nichols-lineage examples, in 2 out of 240 contemporary UK patients. This suggests that some sublineages may truly be rare, while the high frequency of other sublineages could reflect either fitness advantages or epidemiological factors such as infecting patients within particularly high-risk sexual networks, allowing these sublineages to expand more successfully. Singleton or private sublineages could reflect insufficient sampling of a country or region, or sampling biases within a country. These sublineages may therefore reflect transmission networks that are either contained within a less internationally mobile demographic, or may reflect transmission networks common in a region that is otherwise poorly sampled (for example, Africa).

Our observation that the well-studied Nichols reference genomes (largely derived from or related to the original Nichols-1912 isolate) form an isolated clade that is not represented in contemporary TPA is important. One possible explanation is that these samples form a distinct clade due to convergent evolution in the rabbit model. However, this clade contains samples that are both extensively and minimally passaged, while other samples passaged in rabbits are distributed throughout the broader phylogeny and are included in three SS14 sublineages (1, 2, 4) and two additional Nichols sublineages (7, 8). This indicates that passage in the rabbit model has not biased other parts of the phylogeny. The majority of Nichols-lineage strains collected before 1988 belong to this clade, and these samples mostly come from a small group of laboratories in the USA. Therefore, it is also tempting to suggest that this reflects a sampling bias for that time period. However, the phylogenetic placement of TPA_AUSMELT-1—isolated in 1977 in Australia^30^, and independently cultured and sequenced—within the same clade contradicts this hypothesis and may suggest that this clade represents the dominant TPA of the period. The complete absence of related genomes in contemporary sampling could represent a decline to becoming a rare or even extinct lineage, and therefore implies that the Nichols reference strain is not representative of contemporary syphilis or even contemporary Nichols-lineage strains.

Our data show that for some sublineages, modern syphilis is a truly global disease with shared lineages, sublineages and indeed nearly identical strains found all over the world. The large expansions of highly related genomes, in particular sublineage 1, represent the bulk of our dataset, and the widespread sharing of major lineages suggests that we have sampled from a series of globally contiguous sexual networks.

Furthermore, we find evidence of a striking change in the genetic diversity and effective population size of TPA genomes, suggestive of a possible population bottleneck occurring between the late 1990s and early 2000s. This was followed by a rapid expansion of certain sublineages, leading to the contemporary TPA population structure. This bottleneck, potentially a consequence of post-HIV safe-sex messaging, persistent antimicrobial prophylaxis in at-risk HIV-positive populations, and possibly HIV-associated mortality, appears to have led to a striking duality in the TPA dominating populations before and after its occurrence. The rapid expansion may be attributed to a relaxation in sexual behaviour following the widespread introduction of highly active antiretroviral therapy. Notably, although macrolide resistance was neither universally distributed throughout the phylogeny nor present in all sublineages, most of the multi-country sublineages were largely macrolide resistant, and this could have also played a role through off-target effects, for example during treatment of other (particularly sexually transmitted) infections^32^. Azithromycin and other macrolides are no longer recommended treatments at any stage of syphilis in the European syphilis management guidelines^33^. A further possibility is that partial host immunity plays a role in sublineage dynamics, as suggested by previous modelling of aggregated national surveillance data^34^ and reported attenuated symptoms on reinfection^35,36^; this will require detailed investigation of well-characterized sexual networks and examination of hypervariable antigenic genes excluded from the core genome used in our phylogenetic analysis.

Our study has a number of limitations. Our samples were collected in an opportunistic manner using residual samples available in regional or national archives. Since our sampling coverage was uneven, with some countries either missing or undersampled, it was not possible to infer the direction of transmission between countries. While sampling was particularly limited in Africa, Asia and South America, we still provide a snapshot of strains from these regions, all of which overlap with the genetic diversity of our more deeply sampled regions (North America and Europe), suggesting that we have captured dominant global lineages. We also show that even deeply sampled countries can harbour rare sublineages, and it is therefore likely that future studies will reveal further novel diversity. Samples collected before the widespread adoption of molecular diagnostics in the early 2000s are limited here, and this is largely influenced by the difficulty in isolating new strains before the recent development of in vitro culture^22^ and the lack of long-term storage of clinical swabs. Most (but not all) older strains come from the USA, and this could mean that we do not accurately reflect the global population structure before 2000. Moreover, because of limited sampling before 2000, historic lineages that lack extant descendants (for example due to extinction) would not be modelled by our Bayesian Skyline, and this could limit our estimates of historic population diversity.

Despite these limitations, our data show that the *T. pallidum* infecting patients today is not the same *T. pallidum* infecting patients even 30 years ago – ancestral sublineages may have become extinct, being replaced by new sublineages that have swept to dominance across the globe with the dramatic upswing in syphilis cases in the USA, the UK and other Western European countries, which were heavily sampled in our study. That such a bottleneck is linked to HIV-related behavioural change during the 1990s rather than the introduction of antibiotics after the Second World War, further supports the importance of sexual behaviour in transmission dynamics. In future work, it would be interesting to integrate epidemiological evidence of sexual networks in purpose-designed cohort studies to explore this further.

## Methods

### Samples

Overall ethical approval for receipt, handling and sequencing of all clinical samples, as well as for use of UK samples collected as part of public health surveillance and for research was granted by the London School of Hygiene and Tropical Medicine Observational Research Ethics Committee (REF No. 16014) and the National Health Service (UK) Health Research Authority and Health and Care Research Wales (UK; 19/HRA/0112). Samples were de-identified and not linked to any personal identifiable information. As no patient contact took place, no change to clinical care occurred, and as the study consisted only of the use of residual DNA from samples that were already routinely collected, patient consent was deemed unnecessary during ethical approval. Ethical approval for sequencing of samples from Belgium was covered by a provision of the Institutional Review Board of the Institute of Tropical Medicine that allows the further characterization of residual patient samples without additional Ethical Committee clearance. In addition, at the Institute for Tropical Medicine outpatient clinic, the patients are informed that their remnant samples may be used; if they do not consent, they have a form to complete (opt out). Samples from Hungary were collected and preserved as part of the routine diagnostics (standard care), and stored at laboratories that have approval for preservation of such and other clinical samples, and no patient identification information was available; accordingly, these samples did not need a separate ethical approval for use in an anonymized manner. Samples from Russia were collected as part of a previous study that involved molecular epidemiology, and this had ethical approval from The State Research Center of Dermatology, Venereology and Cosmetology of The Russian Ministry of Health (SRCDVC), Moscow, Russia. Samples from South Africa were collected as part of a study on the impact of episodic acyclovir therapy on ulcer duration and HIV shedding from genital ulcers in men, and ethical approval was granted by the Human Research Ethics Committee of the University of the Witwatersrand in South Africa (Clearance Certificate Nos. M040548 and M10201). All participants gave permission to store samples for future testing for infectious diseases. Zimbabwe samples were collected as part of the Zimbabwe STI Etiology Study, which had a provision for specimen storage and future studies, and the consent form had a specific opt-in/opt-out addendum for specimen storage and future studies. It also specifically asked for consent to have specimens shipped to the National Institute for Communicable Diseases in South Africa. The protocol and consent forms were approved by the Research Council and Medical Research Council of Zimbabwe. Samples from Canada (British Columbia and Alberta) were collected as part of public health surveillance, were de-identified before transfer between labs and were deemed exempt from requiring additional ethical approval. Samples from Australia were covered by Human Research Ethics Committee approval, and this approval included a waiver to obtain individual informed consent that was consistent with the requirements outlined in the Australian National Health and Medical Research Council National Statement. For samples from Spain, all the patients enrolled in the study provided written consent for collection of an additional ulcer swab and/or whole blood specimen to perform the TPA molecular studies. Institutional Review Board approval PR(AG)297/2014 was obtained from the Ethics Committee of Vall d’Hebron Research Institute. An amendment was also approved to allow WGS. For samples from Ireland, the study was approved by the ethics board of St James’s Hospital and Tallaght Hospital, and this included approval for molecular analyses.

Novel samples from Australia (Brisbane, Melbourne), Belgium (Antwerp), Canada (Alberta, British Columbia), Hungary (National collection), Ireland (Dublin), Russia (Tuva Republic), South Africa (Johannesburg), Spain (Barcelona), Sweden (National collection), the UK (National collection) and Zimbabwe (3 regions) were sequenced directly from genomic DNA extracted from clinical patient swabs or biopsies, typically utilizing de-identified residual diagnostic samples that were further pseudonymized before analysis. Additional novel samples from Australia (Melbourne), Haiti and the USA (6 cities) were sequenced from historic freezer archives after previous passage in the rabbit model^4^.

DNA extracts were quantified by quantitative real-time PCR (qPCR; TPANIC_0574) as previously described^4^, and grouped into pools of either 32 or 48 with similar (within 2 C_T_) treponemal load, with high-concentration outlier samples diluted as necessary. We added 4 μl pooled commercial human genomic DNA (Promega) to all samples to ensure total genomic DNA > 1 μg per 35 μl, which was sufficient for library prep.

### Sequencing

Extracted genomic DNA was sheared to 100–400 bp (mean distribution 150 bp) using an LE220 ultrasonicator (Covaris). Libraries were prepared (NEBNext Ultra II DNA Library preparation kit, New England Biolabs) using initial adaptor ligation and barcoding with unique dual indexed barcodes (Integrated DNA Technologies). Dual indexed samples were amplified (6 cycles of PCR, KAPA HiFi kit, Roche), quantified (Accuclear dsDNA Quantitation Solution, Biotium), then pooled in pre-assigned groups of 48 or 32 to generate equimolar pools on the basis of total DNA concentration. Pooled DNA (500 ng) was hybridized using 120-mer RNA baits (SureSelect Target enrichment system, Agilent technologies; bait design ELID ID 0616571)^4,37^. Enriched libraries were sequenced on Illumina HiSeq 4000 to generate 150 bp paired-end reads at the Wellcome Sanger Institute (UK) as previously described^38^. For one rabbit-passaged sample from Melbourne, Australia (TPA_AUSMELT-1)^30^, genomic DNA extracted from historically archived tissue lysate was sequenced on Illumina NextSeq 500 (150 bp paired-end reads, Nextera DNA Flex libraries) without any previous enrichment to an estimated 1 Gb per sample at the Doherty Institute (Australia).

### Sequence analysis and phylogenetics

We filtered *Treponema* genus-specific sequencing reads using the full bacterial and human Kraken 2 (ref. ^39^) v2.0.8 database (March 2019), followed by trimming with Trimmomatic^40^ v0.39 and downsampling to a maximum of 2,500,000 using seqtk v1.0 (available at https://github.com/lh3/seqtk) as previously described^4^. For publicly available genomes, raw sequencing reads were downloaded from the European Nucleotide Archive and subjected to the same binning and downsampling procedure. For five public genomes (Supplementary Data 1), raw sequencing reads were not available; for these we simulated 150 bp paired-end perfect reads at 50X coverage from the RefSeq closed genomes using Fastaq v3.17.0 (available at https://github.com/sanger-pathogens/Fastaq).

For phylogenomic analysis, we mapped *Treponema*-specific reads to a custom version of the SS14_v2 reference genome (NC_021508.1) after first masking 12 repetitive Tpr genes (Tpr A–L), two highly repetitive genes (arp, TPANIC_0470) and five FadL homologues (TPANIC_0548, TPANIC_0856, TPANIC_0858, TPANIC_0859, TPANIC_0865) using bedtools^41^ v2.29 maskfasta (positions listed in Supplementary Data 2). We mapped prefiltered sequencing reads to the reference using BWA mem^42^ v0.7.17 (MapQ ≥ 20, excluding reads with secondary mappings), followed by indel realignment using GATK v3.7 IndelRealigner, de-duplication with Picard MarkDuplicates v1.126 (available at http://broadinstitute.github.io/picard/), and variant calling and consensus pseudosequence generation using samtools^43^ v1.6 and bcftools v1.6, requiring a minimum of two supporting reads per strand and five in total to call a variant, and a variant frequency/mapping quality cut-off of 0.8. Sites not meeting our filtering criteria were masked to ‘*N*’ in the final pseudosequence. After mapping and pseudosequence generation, we repeated the masking of the 19 genes on the final multiple sequence alignment using ‘remove_block_from_aln.py’ available at https://github.com/sanger-pathogens/remove_blocks_from_aln/ to ensure sites originally masked in the reference were not inadvertently called with SNPs due to mapped reads overlapping the masked region. These 19 regions of recombination and genomic uncertainty due to gene orthology or repetitive regions^3,4,6^ accounted for 30,071 genomic sites (Supplementary Data 2).

For basic lineage assignment of genomes, we excluded sequences with >75% genomic sites masked to ‘*N*’. A SNP-only alignment was generated using snp-sites^44^ v 2.5.1, and a maximum likelihood phylogeny was calculated on the variable sites using IQ-Tree^28^ v1.6.10, inputting missing constant sites using the ‘-fconst’ argument, and using a general time reversible substitution model with a FreeRate model of heterogeneity^45^ and 1,000 UltraFast Bootstraps^46^.

For fine-scale analysis of high-quality genomes, we excluded sequences with >25% genomic sites masked to ‘*N*’ (that is, >75% genomic sites passing filters at >5X and not masked). We used Gubbins^27^ v2.4.1 (20 iterations) to generate recombination-masked full genome length and SNP-only alignments. Gubbins^27^ identified 19 further putative regions of recombination affecting 2.1% of genomic sites (*n* = 23,567) and 27 genes (listed in Supplementary Data 2), meaning we masked a maximum of 4.7% (53,638 sites) of the genome over the 38 regions. We used IQ-Tree on the SNP-only alignment containing 901 variable sites, inputting missing constant sites using the ‘-fconst’ argument, and allowing the built-in model test to infer a K3Pu+F+I model and 10,000 UltraFast bootstraps.

To cluster genomes, we initially performed joint ancestral reconstruction^47^ of SNPs on the phylogeny using pyjar v0.1.0 (available at https://github.com/simonrharris/pyjar) and used this to determine phylogenetic clusters with a 10 SNP threshold in rPinecone^29^ v0.1.0 (available at https://github.com/alexwailan/rpinecone). We further investigated phylogenetic clustering by using IQ-tree to generate 100 standard non-parametric bootstraps on the maximum likelihood phylogeny and used the resulting 100 trees as independent inputs to rPinecone, as described in the rPinecone manuscript^29^. We used the hierarchical clustering ‘hclust’ algorithm in R^48^ to group rPinecone clusters and evaluated different proportions of trees supporting clusters against the phylogeny (Supplementary Fig. 2).

To assess the impact of missing sites in the multiple sequence alignments used to construct our phylogenies, we subsampled the recombination-masked multiple sequence alignment to only include 301 genomes with <5% of genomic positions masked to ‘*N*’, and repeated the maximum likelihood analysis using IQ-Tree. We converted the resulting phylogeny to an ultrametric tree using phytools^49^ v0.7-47; comparison with the ultrametric tree of our fine-scale analysis of 528 genomes in a tanglegram indicated that the underlying topology and sublineage clusters were the same (Supplementary Fig. 7), except for the low-diversity sublineage 1 region. To assess the impact of mapping to an alternative reference genome, we also mapped the 528 genomes to the Nichols_v2 reference (CP004010.2), repeating the recombination masking and phylogenetic reconstruction as described above. Comparison of the resulting Nichols-mapped ultrametric tree to our SS14-mapped tree in a tanglegram also indicated equivalent topology and clustering of sublineages (Supplementary Fig. 8), with the exceptions of sublineage 1 (which has low diversity and thus low support for within-group topology) and sublineage 6 (which diverges from other strains close to the root of TPA and appears on either the Nichols- or SS14-lineage side of different midpoint rooted trees).

For temporal analysis, our dataset was too large for robust model testing of all genomes, so we stratified our dataset by sublineage and country, then used the random sampler in R^48^ to select a maximum of five genomes from each strata, plus all singleton strains, yielding a dataset of 138. We extracted the sequences from the multiple sequence alignment using seqtk and the subtree from our broader phylogeny using ape^50^ v5.4.1 ‘keep.tip’. Root-to-tip distance analysis from this subtree showed a correlation of 0.327 and R^2^ of 0.11 (Supplementary Fig. 9), and we proceeded to BEAST analysis. We initially ran BEAST^31,51^ v1.8.4 in triplicate on our recombination-masked SNP-only alignment containing 592 variable sites, correcting for invariant sites using the ‘constantPatterns’ argument, using both a Strict Clock model^52^ (starting rate prior 1.78 × 10^−7^) and an Uncorrelated Relaxed Clock model^53^, with HKY substitution model^54^ and diffuse gamma distribution prior^55^ (shape 0.001, scale 1,000) over 100 million Markov Chain Monte Carlo (MCMC) cycles with 10 million cycle burn-in. We evaluated constant, relaxed lognormal, exponential and Bayesian Skyline (10 categories) population distributions^56^. All MCMC chains converged with high effective sample sizes, and on inspection of the marginal distribution of ‘ucld.stdev’, we could not reject a Strict Clock. We used the marginal likelihood estimates from the triplicate BEAST runs as input to Path Sampling and Stepping Stone Sampling analysis^57,58^, and this suggested that the Strict Clock with Bayesian Skyline was the optimal model for this dataset (Supplementary Fig. 10), with an inferred molecular clock rate of 1.23 × 10^−7^ substitutions per site per year. This is consistent with previous molecular clock rate estimates for TPA^3,4^, but we note that inclusion of recombinogenic or hypervariable sites outside the clonal frame (masked in this study) would be expected to increase this rate. To ensure that our findings were not artefactual to the downsampled dataset, we re-stratified the dataset by sublineage, country and year, selecting a maximum of 3 genomes from each stratum, plus all singleton strains, yielding a dataset of 168 genomes with 466 variable sites. We ran BEAST on this new subsampled dataset using the optimal Strict Clock (with starting rate prior of 1.23 × 10^−7^, inferred from the previous analysis) with Bayesian Skyline from over 100 million MCMC cycles, with the equivalent results (Supplementary Fig. 11).

To evaluate the temporal dynamics of sublineages, we tested the temporal signal for the 4 largest sublineages 1, 2, 8 and 14 (Supplementary Fig. 4). Sublineage 14 had poor temporal signal and was excluded from further analysis. We performed independent BEAST analyses on the remaining sublineage multiple sequence alignments using the optimal Strict Clock model with Bayesian Skyline (10 population groups) described above, evaluating 3 independent chains of 200 million cycles for each.

To analyse the full dataset (*n* = 520 after excluding heavily passaged samples or those with uncertain collection dates, 883 variable sites) after evaluating temporal signal (Supplementary Fig. 12), we initially attempted to reproduce our model in BEAST 1.8.4, but this proved unachievable with our local implementation and computing arrangements. To analyse the full dataset, we therefore reconstructed the optimal BEAST v1.8.4 model (Strict Clock with reference rate prior of 1.23 × 10^−7^ substitutions per site per year, HKY substitution model^54^, Coalescent Bayesian Skyline distribution with 10 populations^54,56^) in a BEAST2^59^ v2.6.3 implementation with BEAGLE^51^ libraries optimized for Graphical Processing Units, analysing the 520 genomes over 500 million MCMC cycles in triplicate. To compare the phylodynamics of the individual Nichols and SS14 lineages, we repeated the BEAST2 analysis described above using multiple sequence alignments specific to Nichols (*n* = 94) and SS14 (*n* = 426); we used Tracer^31^ v1.7.1 to extract Bayesian Skyline distributions and lineage accumulation for plotting in R.

To further confirm the temporal signal in our full 520-genome tree, we used the TIPDATINGBEAST^60^ v1.1-0 package in R to perform a date randomization test, generating 20 new datasets with randomly re-assigned dates from the original xml file and conditions – BEAST2 analysis using the same prior conditions found no evidence of temporal signal in these replicates, indicating that the signal in our tree was not found by chance (Supplementary Fig. 13).

We used logcombiner v2.6.3 with a 10% burn-in to generate consensus log and tree files, resampling 100,000 states for the full 520 sample analysis, and treeannotator v1.8.4 to create median maximum credibility trees. We generated Bayesian Skyline and lineage accumulation plots using the combined log and tree files in Tracer v1.7.1^31^, exporting the data for subsequent plotting in R. To evaluate the posterior distribution of population expansion times, we used the script ‘population_increase_distribution_BEAST.py’ (available at https://github.com/chrisruis/tree_scripts commit: 2463656e329e3f25ec6dd13c86c64ad163525ae0), which uses the BEAST log and tree files to identify the first increase in relative genetic diversity from the PopSizes columns and the date of this increase using the corresponding number of nodes in the GroupSizes columns and the node heights in the respective tree. We required a 2-fold population expansion (defined by setting ‘-p’ to 100). The script outputs the proportion of trees in the posterior distribution that support an increase in relative genetic diversity, along with the distribution of expansion dates, which we plotted in R^48^. We repeated this analysis using the script ‘population_change_support_BEAST.py’ (available at https://github.com/chrisruis/tree_scripts), which looks for an increase or decrease of effective population size within a defined window, testing for supported start dates of a 2-fold population decline or expansion between 1990 and 2015.

For analysis of genetic changes between common ancestral nodes in our phylogeny, we performed ancestral reconstruction of the full 528-sample maximum likelihood alignment and tree using TreeTime^61^ v0.7.4. We extracted SNPs from the resulting multiple sequence alignment using snp-sites, functionally annotated variants using SnpEff^62^ v4.3 with the most recent National Center for Biotechology Information annotation for the NC_021508.1 SS14 reference genome (June 2021), and imported data into R^48^ v3.6.0 for analysis using vcfR^63^ v1.12.0. We selected the common ancestral nodes leading to contemporary SS14 and Nichols lineages (Supplementary Fig. 6), and used R to extract annotated variants that differed between the relevant nodes from our variant call file (VCF).

For comparison of TPA sublineage trends with national syphilis rates, we downloaded and plotted publicly available incidence data for England (https://www.gov.uk/government/statistics/sexually-transmitted-infections-stis-annual-data-tables) and British Columbia (http://www.bccdc.ca/health-professionals/data-reports/sti-reports).

Macrolide resistance alleles were inferred using the competitive mapping approach previously described^4,38^ (available at https://github.com/matbeale/Lihir_Treponema_2020/competitive_mapping_Treponema23S–mod.sh commit: 044b29ce29ada81e4f7cb0318301e97e1d5a8d55). To infer pairwise SNP distances between samples, we used pairsnp v0.1.0 (available at https://github.com/gtonkinhill/pairsnp commit: 0acddba060cc076946dab9969a95ab3c21f110fb). We constructed networks of minimum pairwise distance and shared lineages in R, and plotted these as heatmaps using ggplot2^64^ v3.3.2. Nucleotide diversity (π) for different clades was inferred from the multiple sequence alignments using EggLib^65^ v3.0.0b21, including variable sites present in at least 5% of genomes. For geospatial analysis, we used the CoordinateCleaner^66^ v2.0-17 package in R to define the centroid position for each country, except for Russia (where we used the centroid of the Tuva Republic) and Mexico (where we used Mexico City). Geographic distances between countries (using the country centroid or location defined above) were determined using the ‘distVincentyEllipsoid’ function from the geosphere^67^ v1.5-10 package. Correlations between pairwise genetic, geographic and temporal distance were inferred using two-sided Pearson’s R Correlation via the ‘cor’ function in R, where we compared ‘real’ correlation with 1,000 bootstraps resampled with replacement to obtain a *P* value. Sample counts were plotted using ggmap^68^ v3.0.0 over maptiles downloaded from Stamen Design (http://maps.stamen.com). All phylogenetic trees were plotted in R using ggtree^69^ v2.5.1. All figures used ggplot2 (ref. ^64^) for plotting and multi-panel figures were constructed using cowplot^70^ v1.1.1.

### Reporting Summary

Further information on research design is available in the Nature Research Reporting Summary linked to this article.

## Supplementary information

Supplementary Information Supplementary Figs. 1–13 and Discussion.

Reporting Summary

Peer Review File

Supplementary Data 1–4 Supplementary Data 1–4 (large spreadsheets, separated into tabs).

## Data Availability

Sequencing reads for all novel genomes have been deposited at the European Nucleotide Archive (https://www.ebi.ac.uk/ena/browser/home) in BioProjects PRJEB28546, PRJEB33181 and PRJNA701499. All accessions, corresponding sample identifiers and related metadata are available in Supplementary Data 1. Map tiles were downloaded from http://maps.stamen.com using the ggmap interface. Publicly available syphilis incidence data are available for England at https://www.gov.uk/government/statistics/sexually-transmitted-infections-stis-annual-data-tables and for British Columbia at http://www.bccdc.ca/health-professionals/data-reports/sti-reports. All sample metadata and intermediate analysis files are available at 10.6084/m9.figshare.14376749 and https://github.com/matbeale/Contemporary_Syphilis_Lineages_2021. The minimum raw datafiles required to construct the Main and Extended Data figures are described in Supplementary Data 4. The fine-scale maximum likelihood phylogeny and metadata are also available for interactive visualization at https://microreact.org/project/xt7AuLJorkyBNHVXL2sF8G/1a515b2c.

## Code availability

The R code used for all statistical analyses and plotting is available in an Rnotebook at 10.6084/m9.figshare.14376749 and at https://github.com/matbeale/Contemporary_Syphilis_Lineages_2021, along with underlying source files.
