# Supplementary material for: Global phylogeny of Treponema pallidum lineages reveals recent expansion and spread of contemporary syphilis
Source: Nat Microbiol. Author manuscript; Available in PMC 2021 Dec 3. (PMC8612932; doi:10.1038/s41564-021-01000-z)
Supplement: Supplementary Information [file EMS137223-supplement-Supplementary_Information.pdf]

Global phylogeny of Treponema pallidum lineages reveals recent expansion and spread of contemporary syphilis

Beale *et al*, 2021

Supplementary Figures

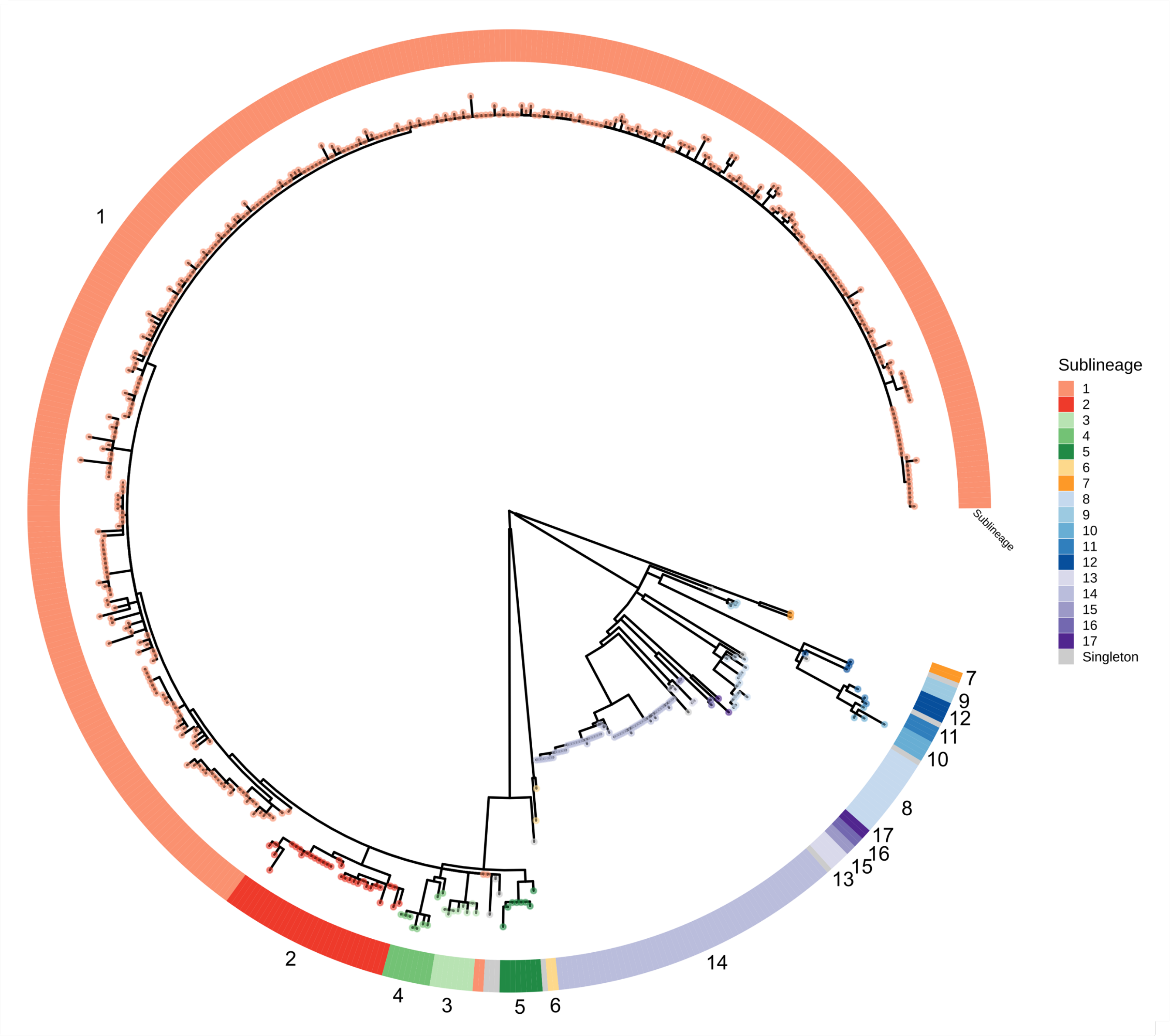

Supplementary Figure 1. Finescale analysis of 528 high quality (>75% reference sites) TPA genomes and sublineages. Recombination masked WGS phylogeny of 528 genomes. Tree tips and coloured strips show sublineage. Note that sublineage 6 diverges from the rest of TPA close to the root, and in this midpoint rooted tree appears on the SS14 side of the root.

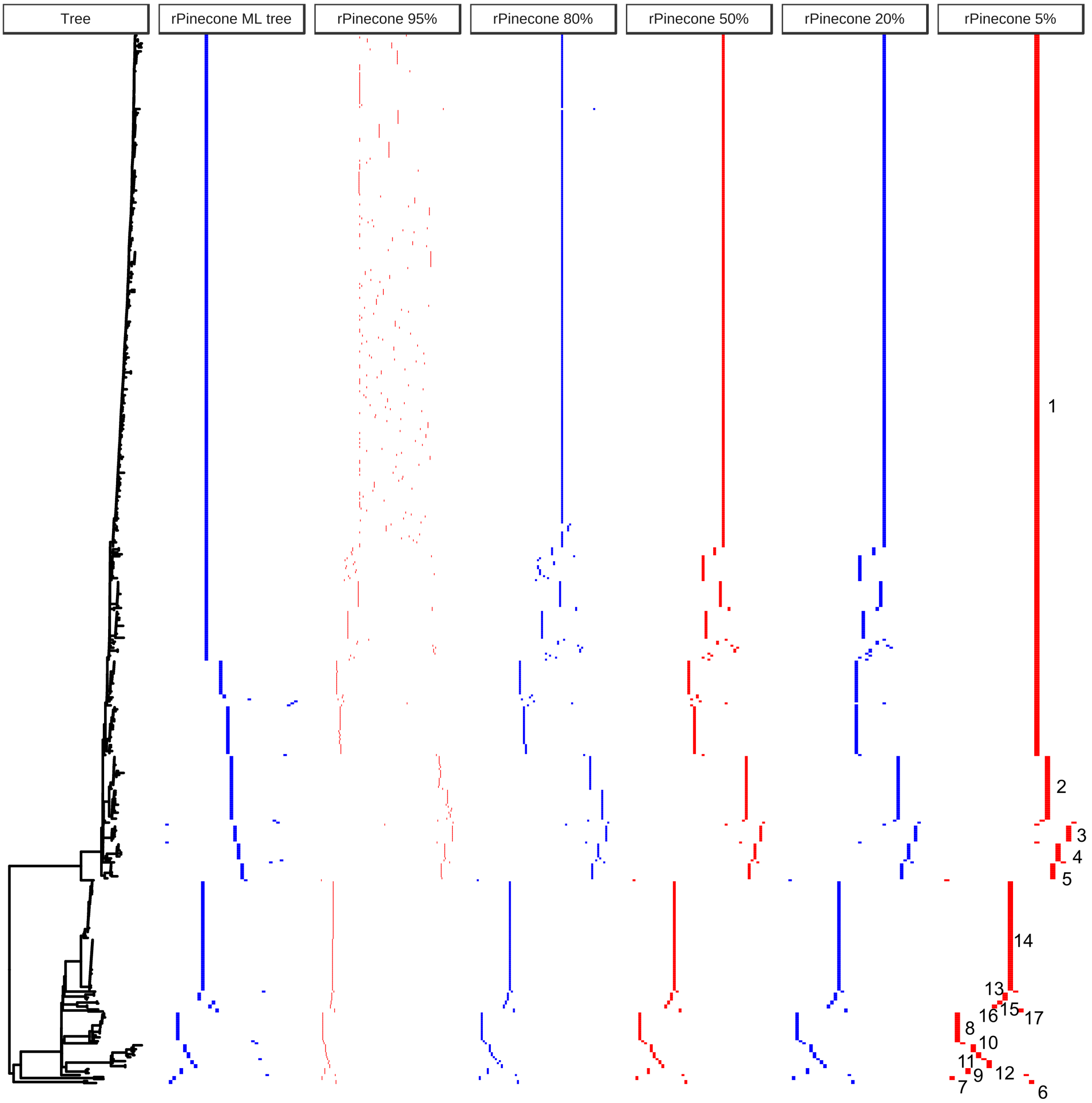

Supplementary Figure 2. Evaluating phylogenomic clustering using bootstrap resampled trees. We generated 100 bootstraps from our finescale analysis of 528 TPA genomes, independently running rPinecone (10 SNP threshold) on each bootstrapped tree. Hierarchical clustering was used to group rPinecone sublineages, and we applied different support thresholds (minimum % of trees remaining) to explore the consistency of sublineages. Nichols-sublineages were all well supported, but some SS14-sublineages lacked support in many bootstraps. To focus on the more stable sublineages we required that at least 5% of the bootstrap replicates supported a cluster. Plot shows maximum likelihood phylogeny, with metadata columns showing cluster assignment along the x-axis for the original maximum likelihood cluster assignment, then allowing for 95%, 80%, 50%, 20% and 5% of bootstrap variation observed. Final sub-lineage assignments are shown against the 5% cluster assignments. Note that non-zero branch lengths were added by IQ-Tree during maximum likelihood tree estimation, leading to an artifactual ladder-like appearance for sublineage 1.

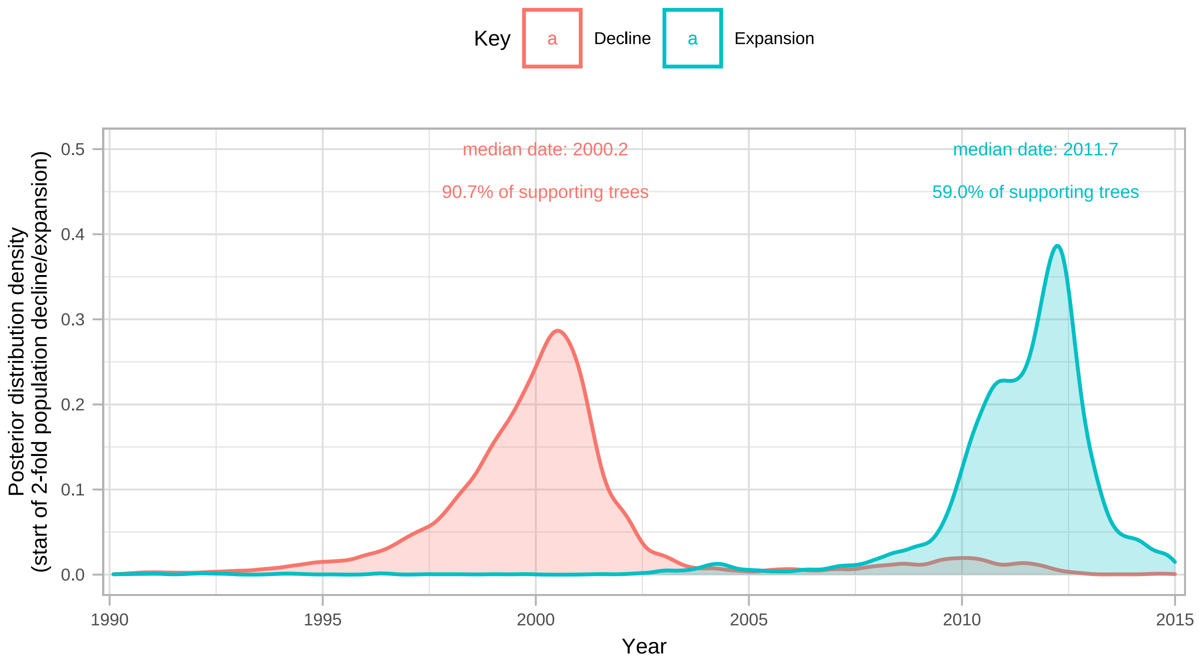

Supplementary Figure 3. Bayesian Skyline analysis of population decline and expansion start dates. Plots show posterior distribution of supporting trees for the start of either a 2-fold decline (pink) or expansion (blue) using a scanning approach within a window of 1990-2015. Analysis provides strong support for a population bottleneck in or around 2000, and moderate support for a subsequent expansion after 2010. Population changes are scaled to the population size averaged over the starting period for each tree. Therefore, if a particular tree already exhibited a decline near the starting timepoint, this may mean this tree does not show expansion, resulting in reduced overall support for expansion.

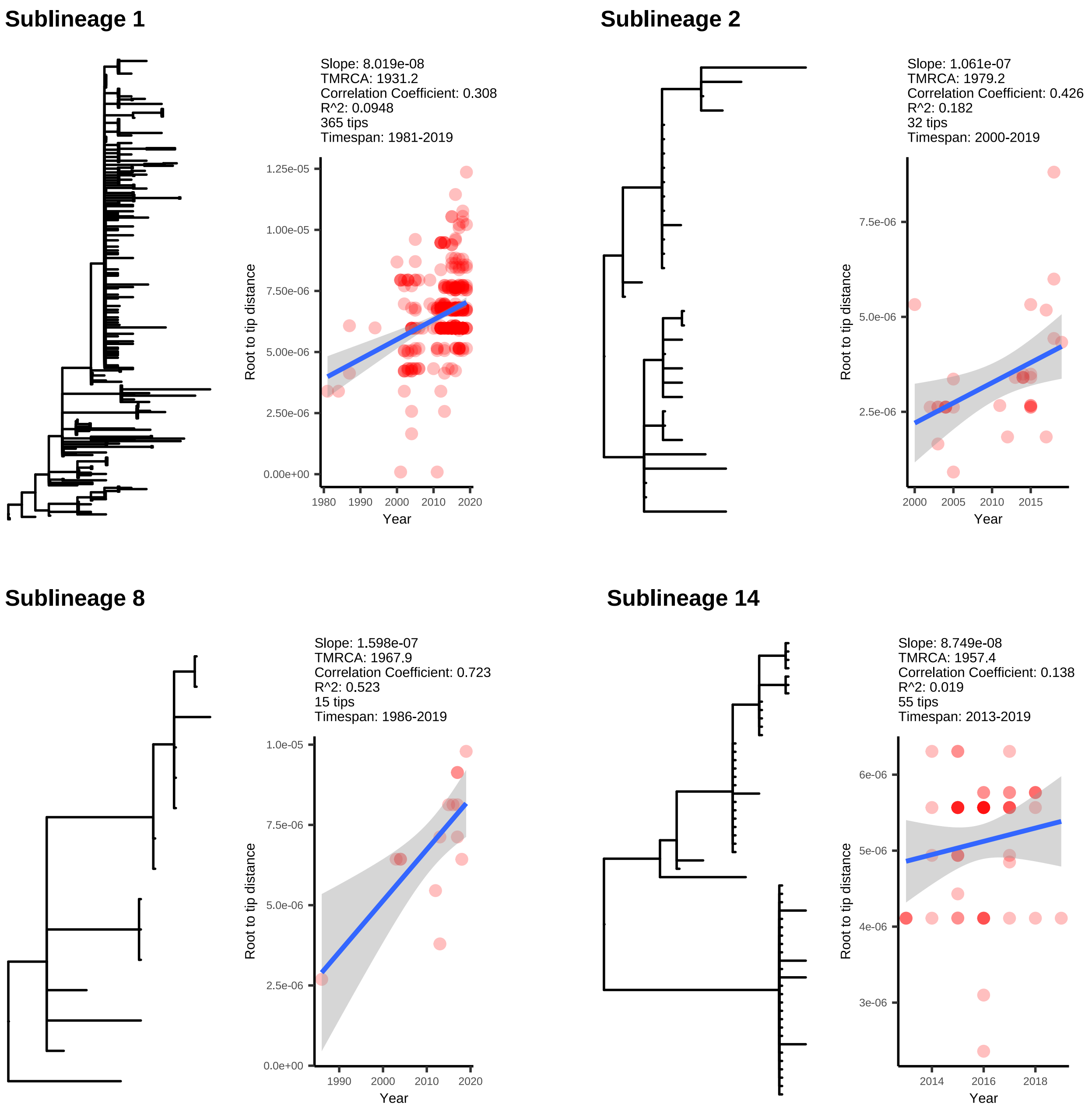

Supplementary Figure 4. Subtrees of major sublineages, with corresponding root-to-tip distance plots. All subtrees showed some evidence of temporal signal, but this was very weak for the recently emerged sublineage 14. Graphs are annotated with slope and time to most recent common ancestor (TMRCA) inferred directly from the maximum likelihood subtree, not BEAST. Line plots show a linear model fitted over the full data range +/- Standard Error.

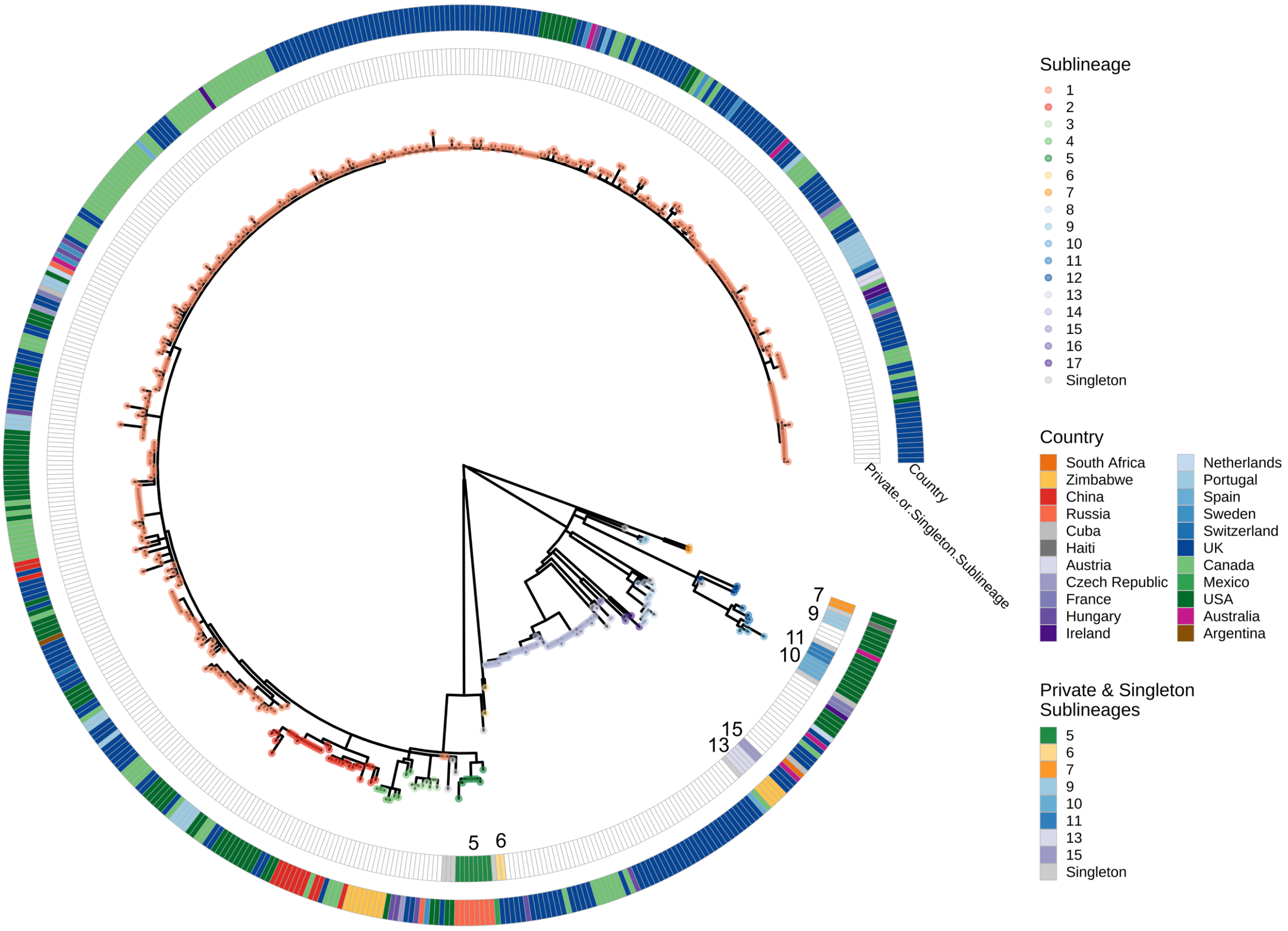

Supplementary Figure 5. Finescale analysis of 528 high quality TPA genomes and sublineages, highlighting private and singleton sublineages. Private and singleton sublineages are nested within the existing diversity of the TPA phylogeny. Tip points indicate sublineage, coloured tracks highlight singletons or private sublineages (with corresponding sublineage number), and country.

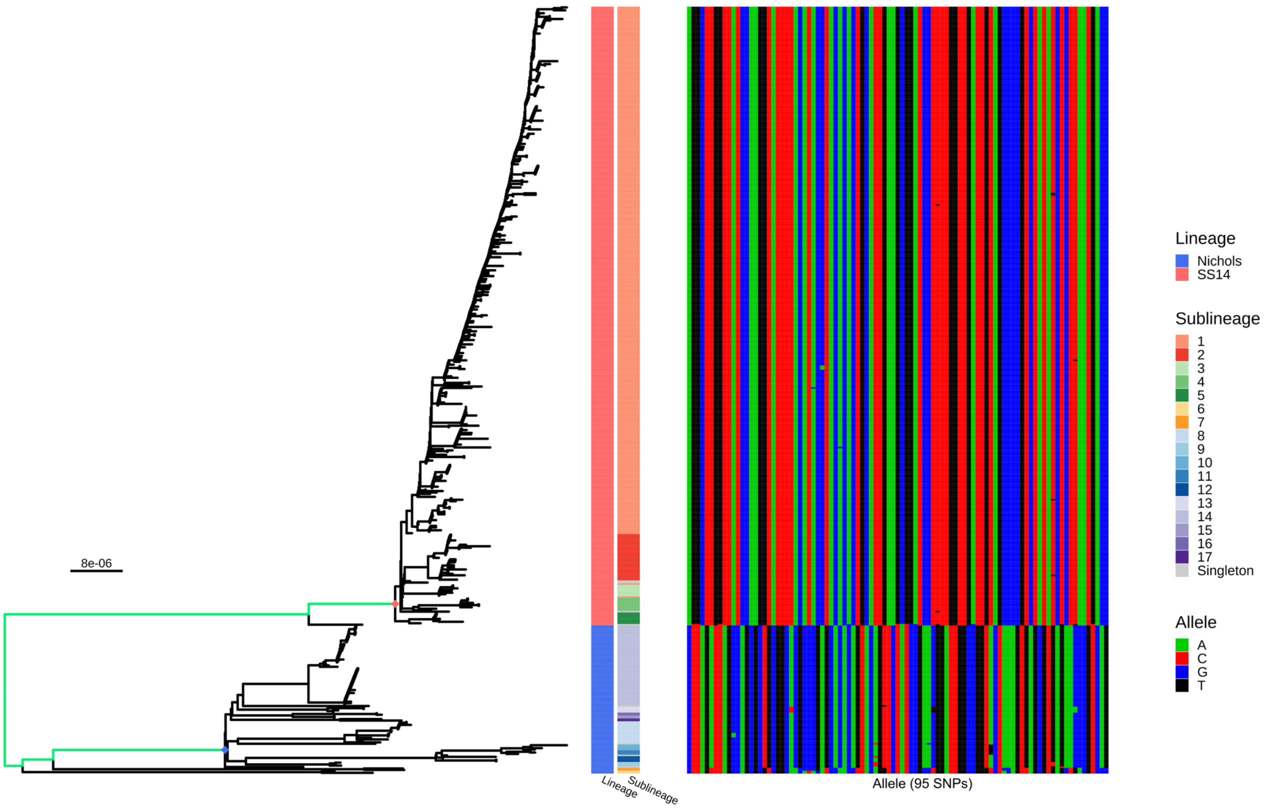

Supplementary Figure 6. Ancestral reconstruction of common ancestral nodes for contemporary Nichols and SS14 clades. Maximum likelihood phylogeny with ancestral reconstruction of node states, highlighting the nodes used for SS14 (all SS14 excluding Mexico_A; pink diamond) and Nichols (all Nichols excluding sublineages 6 and 7; blue diamond). Coloured strips indicate Lineage, Sublineage, and the Allele (SNP) at 95 discriminatory sites (see Supplementary Data 3 for details of SNPs). The branches which separate the two nodes used are shown in green. Note that in this midpoint rooted tree, sublineage 6 is placed on the Nichols side of the root.

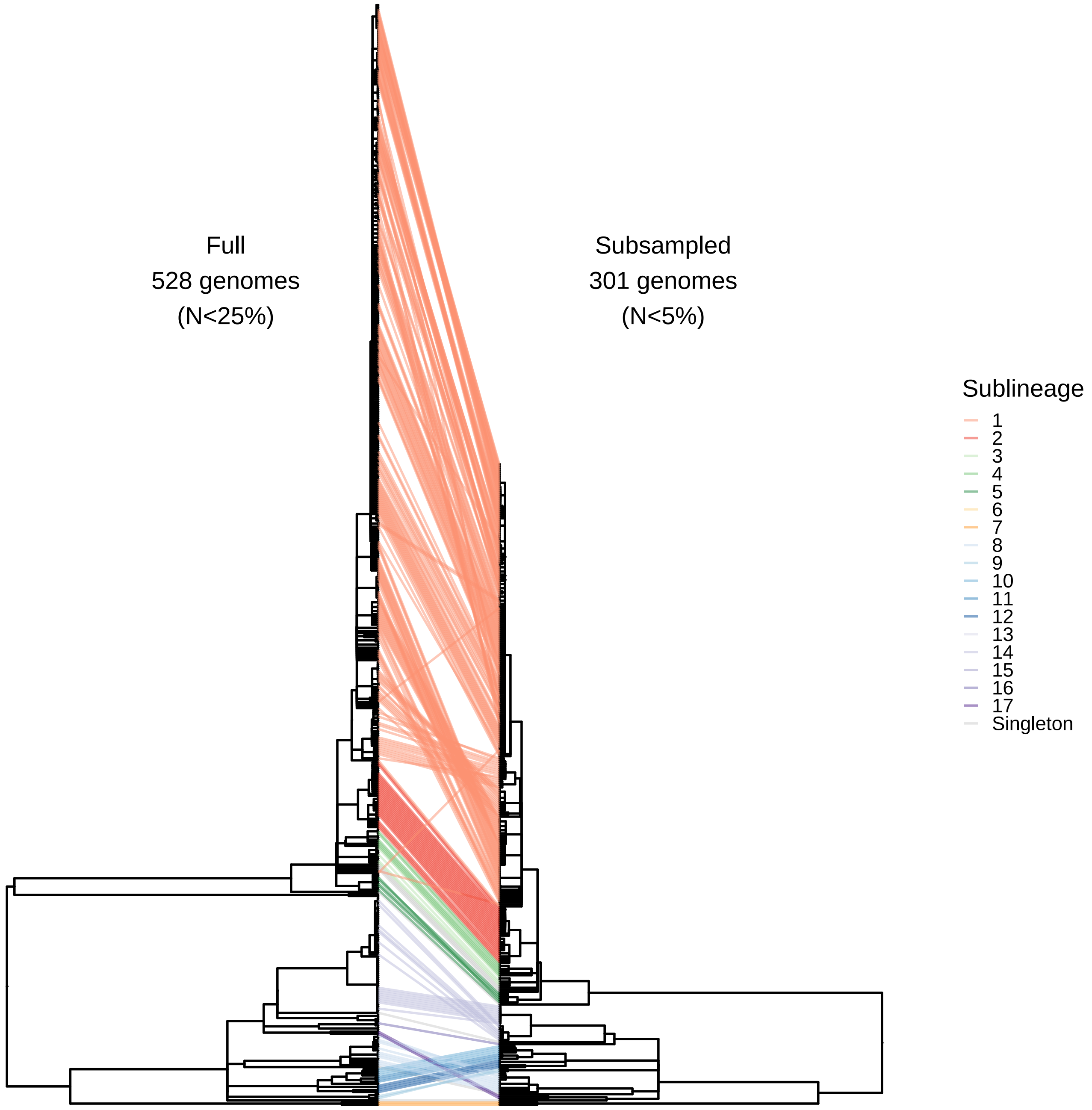

Supplementary Figure 7. Tanglegram comparing tree topology and clustering of sublineages based on proportion of genome masked to ‘N’ shows equivalent clustering. Comparison between ultrametric trees using the full 528 genome Maximum Likelihood analysis (maximum 25% sites masked to ‘N’; left) and a subsampled dataset of 301 genomes (maximum of 5% sites masked to ‘N’; right).

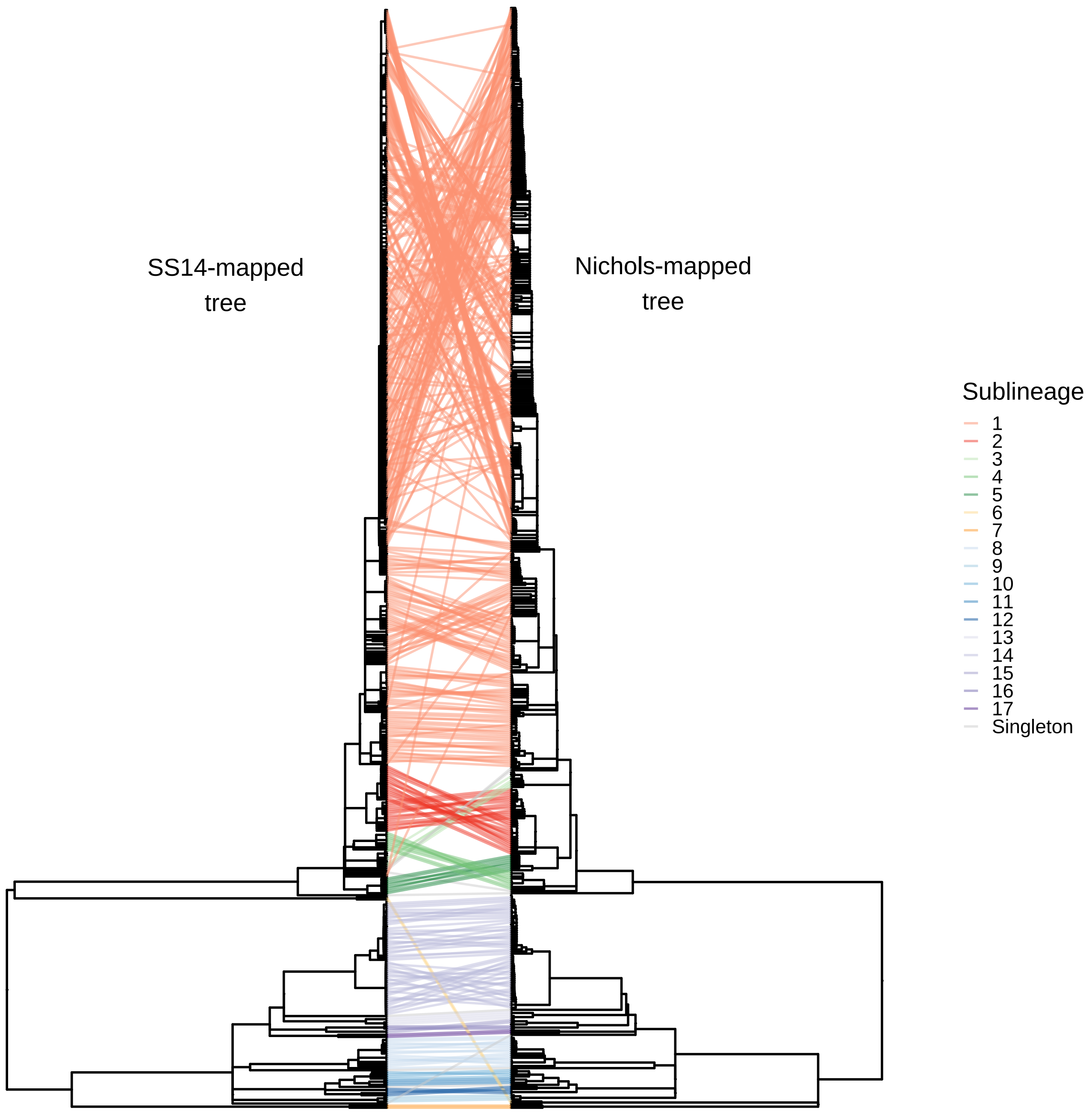

Supplementary Figure 8. Tanglegram comparing tree topology and clustering of sublineages based mapping to either the SS14_v2 reference genome or the Nichols_v2 reference genome shows equivalent clustering. Comparison between ultrametric trees using the full 528 genome post-gubbins Maximum Likelihood analysis mapped to SS14 (NC_021508.1; left) and the same analysis repeated for the Nichols reference (CP004010.2; right).

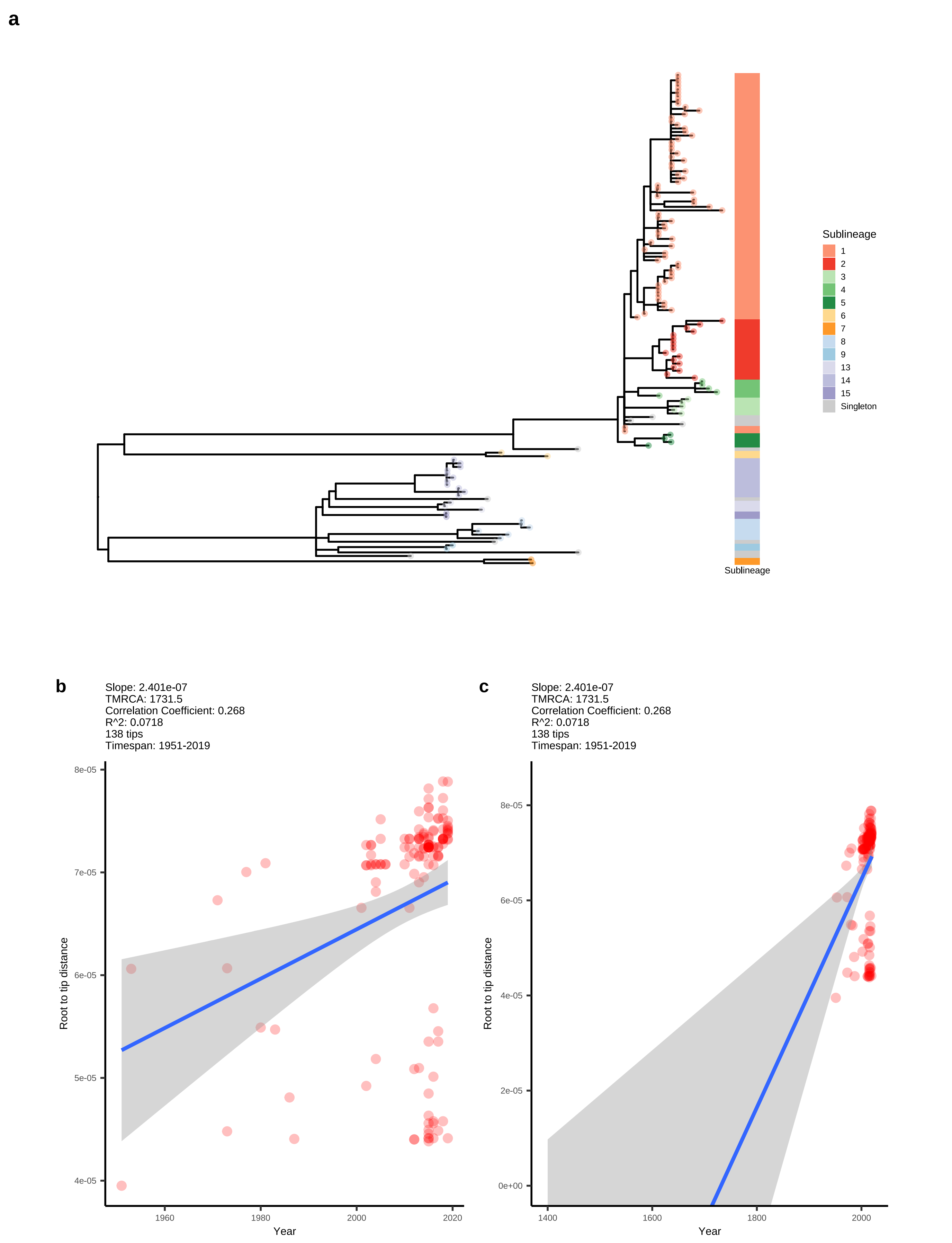

Supplementary Figure 9. Maximum Likelihood phylogeny of 138 representatively subsampled genomes.

A- Maximum likelihood phylogeny of 138 genomes randomly sampled to be representative of sublineage

and country. B- Scatterplot showing root-to-tip distance against collection date, illustrating temporal signal in the dataset. C- Expanded version of B, showing regressed x-intercept. Line plots show a linear model fitted over the full data range +/- Standard Error.

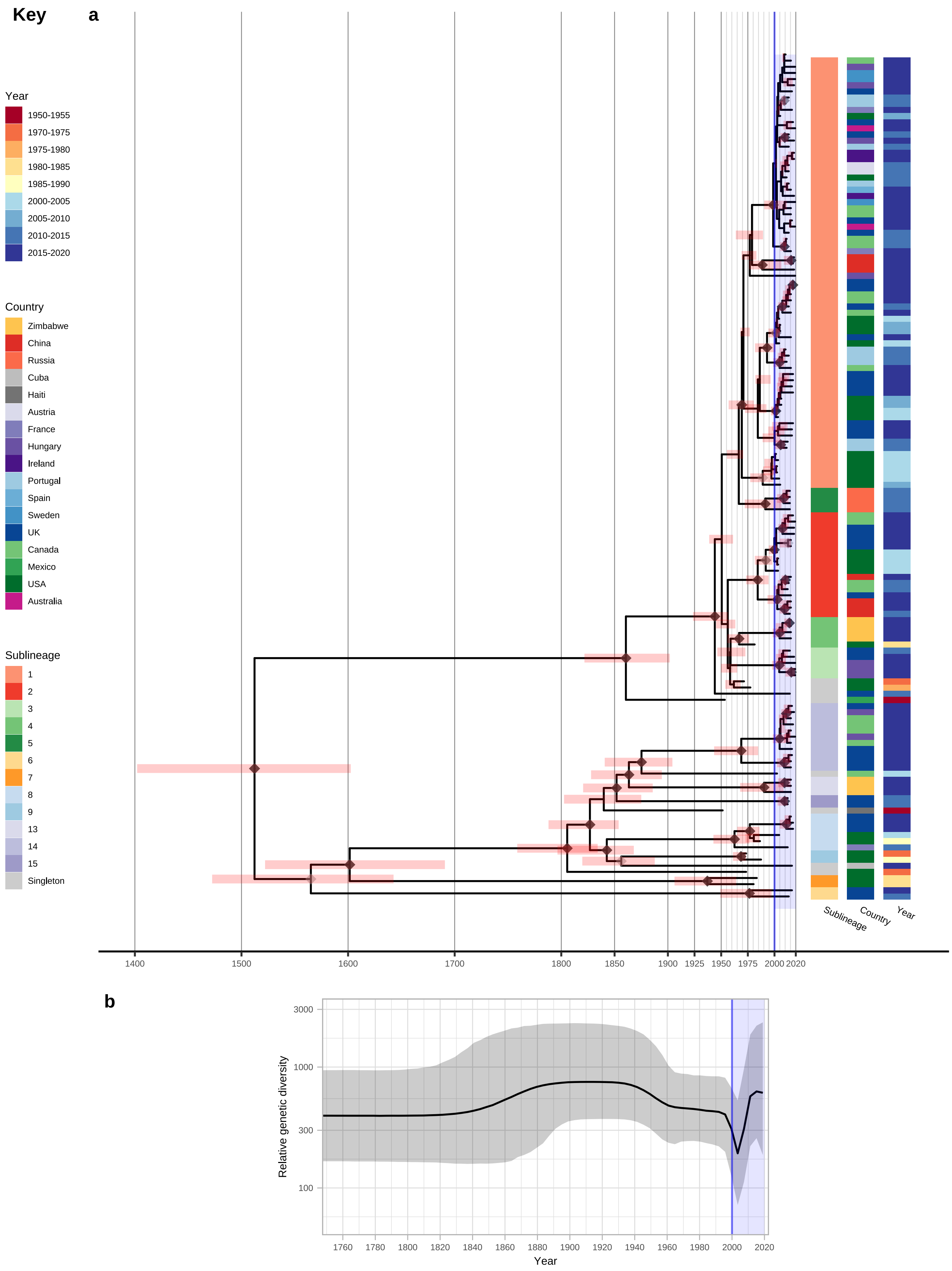

Supplementary Figure 10. Bayesian maximum credibility phylogeny of 138 representative genomes shows population contraction during the 1990s, followed by rapid expansion from the early 2000s

onwards. A- Time-scaled phylogeny of 138 genomes randomly sampled to be representative of sublineage, country and collection year. Coloured tracks indicate sublineage, country and collection year. Node points are shaded according to posterior support (black ≥96%, dark grey >91%, light grey >80%). Red bars on nodes indicate 95% Highest Posterior Density intervals. Blue line and shaded area highlights post-2000 expansion of lineages. B- Bayesian Skyline plot shows decline of effective population size after the second world war, flattening in the 1960s, followed by a sharp decline and rapid reemergence during the 1990s and 2000s.

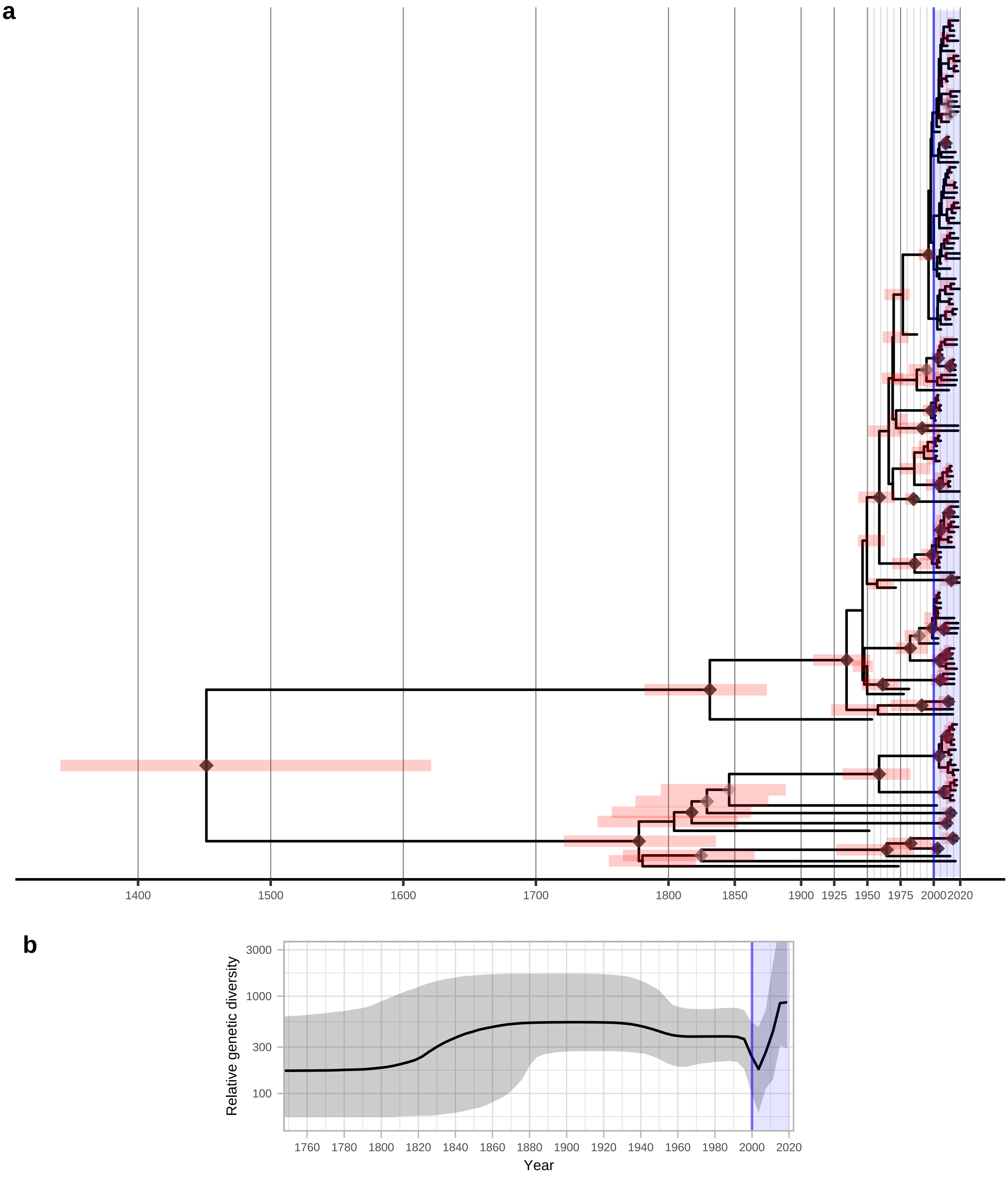

Supplementary Figure 11. Secondary BEAST analysis of 168 separately subsampled representative genomes. A- Time-scaled phylogeny of 168 genomes randomly sampled to be representative of sublineage, country and collection year. Node points are shaded according to posterior support (black ≥96%, dark grey >91%, light grey >80%). Red bars on nodes indicate 95% Highest Posterior Density intervals. Blue line and shaded area highlights post-2000 expansion of lineages. B- Bayesian Skyline plot shows decline of effective population size after the second world war, flattening in the 1960s, followed by a sharp decline and reemergence during the 1990s and 2000s, indicative of a sharp bottleneck.

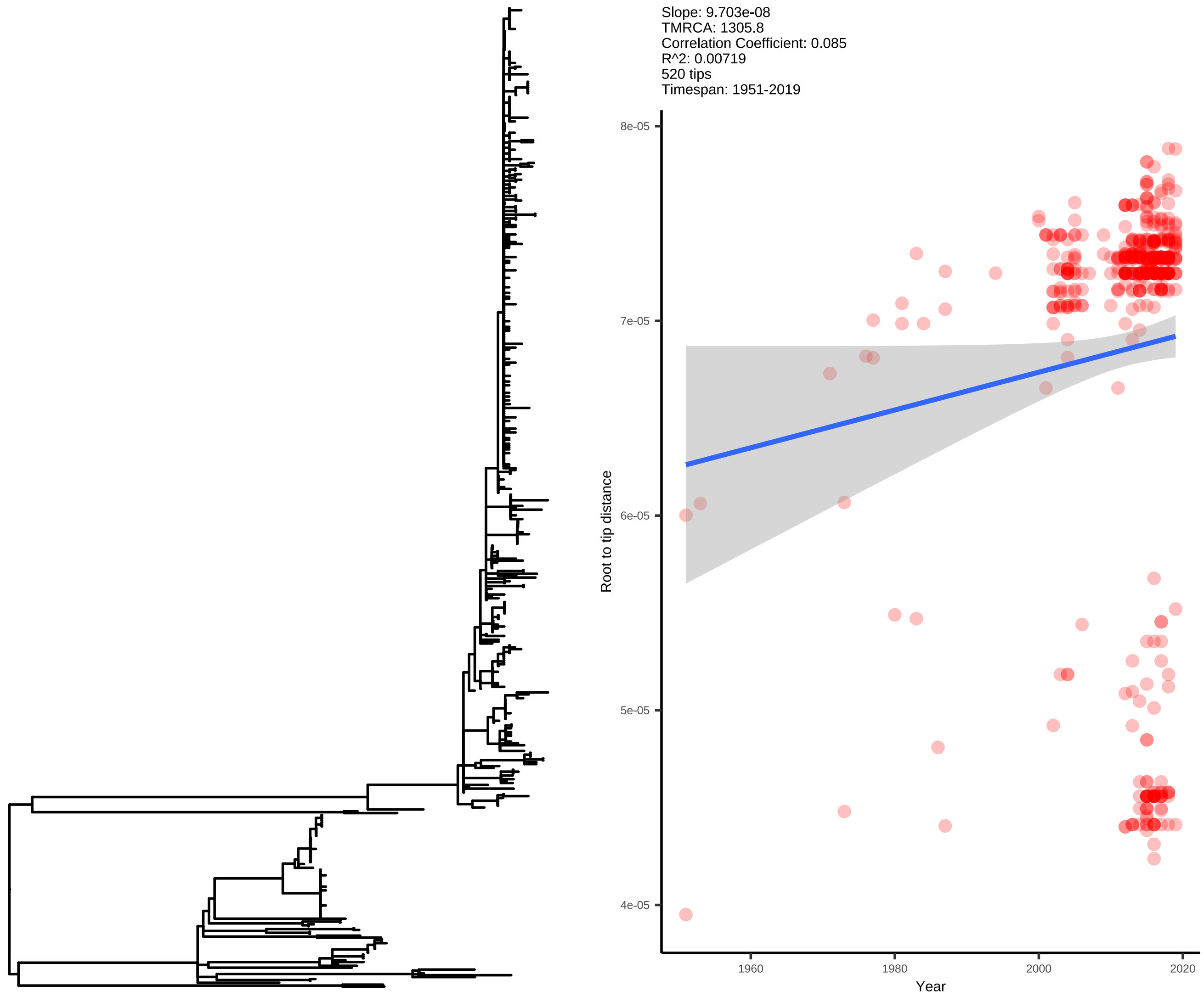

Supplementary Figure 12. Maximum likelihood tree of 520 TPA genomes with minimal passage and robust collection dates, with corresponding root-to-tip distance plot. Within the full tree, the temporal signal was weaker than in our subsampled dataset, but still plausible, given our prior analyses. Graphs are annotated with slope and time to most recent common ancestor (TMRCA) inferred directly from the maximum likelihood subtree, not BEAST. Line plots show a linear model fitted over the full data range +/- Standard Error.

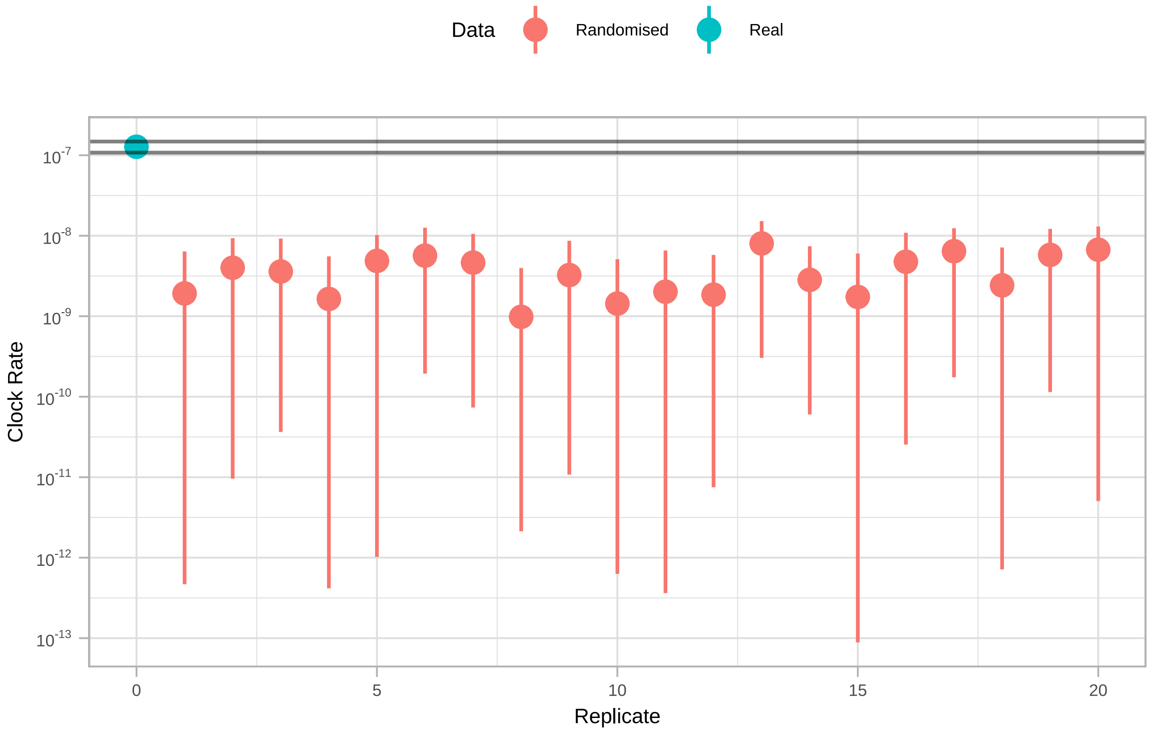

Supplementary Figure 13. Date Randomisation Test for full BEAST2 dataset confirms the temporal signal in the true dataset compared to 20 resampled datasets with randomly reassigned tipdates. Data presented shows the median (point) +/- 95% Highest Posterior Density (HPD; line range) from 21 independent runs (n=5x10^8 Markov Chain Monte Carlo cycles, with first 5 x10^7 cycles removed as burnin). The median clock rate for the real dataset was 1.27 x10^{-7} substitutions/site/year, while all randomly assigned datasets gave substantially lower clock rates; the highest median clock rate for the randomized datasets was 8.02 x10^{-9} substitutions/site/year. Real sample (blue), randomized samples (pink).

Supplementary Data 1. Metadata and read accessions for all samples included in this study

Supplementary Data 2. Genomic regions masked due through prefiltering or recombination analysis.

Supplementary Data 3. Discriminatory SNPs between common ancestral sequences of contemporary Nichols and SS14 lineages, with functional annotation calls.

Supplementary Data 4. List of raw data files supporting Main and Extended Figures.

Supplementary Discussion

There have been some documented reports of clinically diagnosed syphilis caused by *Treponema pallidum* subspecies *endemicum* (TEN)[1,2]. In our study, most novel genomes were clinically diagnosed and confirmed by diagnostic PCRs that do not discriminate between subspecies, yet we found only TPA. Therefore, although we cannot rule out that TEN causes syphilis, due to the limits of our sampling framework, our data suggest TEN is not a major contributor to the burden of syphilis in any of our well sampled countries.

References

1. Noda, A. A. *et al.* Bejel in Cuba: molecular identification of *Treponema pallidum* subsp. *endemicum* in patients diagnosed with venereal syphilis. *Clin. Microbiol. Infect.* **24**, 1210.e1-1210.e5 (2018).

2. Kojima, Y., Furubayashi, K., Kawahata, T., Mori, H. & Komano, J. Circulation of Distinct *Treponema pallidum* Strains in Individuals with Heterosexual Orientation and Men Who Have Sex with Men. *J. Clin. Microbiol.* **57**, e01148-18 (2019).
