# Supplementary material for: Global phylogeny of Treponema pallidum lineages reveals recent expansion and spread of contemporary syphilis
Source: Nat Microbiol. 2021 Nov 24;6(12):1549–60. doi: 10.1038/s41564-021-01000-z (PMC8612932; doi:10.1038/s41564-021-01000-z)
Supplement: Supplementary file 2 [file 41564_2021_1000_MOESM2_ESM.pdf]

nature portfolio

Corresponding author(s): Mathew A Beale

Last updated by author(s): Oct 8, 2021

Reporting Summary

Nature Portfolio wishes to improve the reproducibility of the work that we publish. This form provides structure for consistency and transparency in reporting. For further information on Nature Portfolio policies, see our Editorial Policies and the Editorial Policy Checklist.

Statistics

For all statistical analyses, confirm that the following items are present in the figure legend, table legend, main text, or Methods section.

n/a	Confirmed	
☐	☒	The exact sample size (*n*) for each experimental group/condition, given as a discrete number and unit of measurement
☒	☐	A statement on whether measurements were taken from distinct samples or whether the same sample was measured repeatedly
☒	☐	The statistical test(s) used AND whether they are one- or two-sided<br>*Only common tests should be described solely by name; describe more complex techniques in the Methods section.*
☐	☒	A description of all covariates tested
☒	☐	A description of any assumptions or corrections, such as tests of normality and adjustment for multiple comparisons
☐	☒	A full description of the statistical parameters including central tendency (e.g. means) or other basic estimates (e.g. regression coefficient) AND variation (e.g. standard deviation) or associated estimates of uncertainty (e.g. confidence intervals)
☒	☐	For null hypothesis testing, the test statistic (e.g. *F*, *t*, *r*) with confidence intervals, effect sizes, degrees of freedom and *P* value noted<br>*Give P values as exact values whenever suitable.*
☐	☒	For Bayesian analysis, information on the choice of priors and Markov chain Monte Carlo settings
☒	☐	For hierarchical and complex designs, identification of the appropriate level for tests and full reporting of outcomes
☐	☒	Estimates of effect sizes (e.g. Cohen's *d*, Pearson's *r*), indicating how they were calculated

Our web collection on statistics for biologists contains articles on many of the points above.

Software and code

Policy information about availability of computer code

Data collection

No software was used for data collection.

Data analysis

Sequence reads were taxonomically classified using Kraken2 v2.0.8 with the full bacterial and human database (2019-03-29). Reads were trimmed using Trimmomatic v0.39 and downsampled using seqtk v1.0. Simulated reads were generated from published assemblies using Fastaq v3.17.0. The SS14 reference genome was masked for known regions of uncertainty/recombination using bedtools v2.29 maskfasta. Sequencing reads were mapped to the reference genome using BWA mem v0.7.17, followed by indel realignment using GATK v3.7, deduplication with Picard MarkDuplicates v1.126, and variant calling with samtools v1.6 and bedtools v1.6 to generate a pseudosequence and multiple sequence alignment. We repeated masking of the known regions using remove_block_from_aln.py (available at https://github.com/sanger-pathogens/remove_blocks_from_aln/). We repeated the same procedures for mapping to the Nichols reference genome.

For phylogenetic analysis, we converted whole genome length multiple sequence alignments to SNP-only alignments using snp-sites v2.5.1 (available at https://github.com/sanger-pathogens/remove_blocks_from_aln/, commit a274ec4e47528a4f1ad7d9445d7a416fe8994397), and also for calculating the invariant site counts. Maximum likelihood phylogenies were calculated using IQ-Tree v1.6.0. For finescale phylogenetic analysis, we used Gubbins v2.4.1 to identify and mask putative regions of recombination. For phylogenetic clustering, we used pyjar v0.1.0 (available at https://github.com/simonrharris/pyjar/, commit 87e93a1c909a6024990a56d3a88dc82627315b27), and rPinecone v0.1.0 (available at https://github.com/alexwailan/rpinecone, commit 36114f5795701340660c141e8269dbeddf53e72a).

For subsampling of the phylogeny, we used ape v5.4.1 and seqtk. For initial BEAST analysis, we used BEAST 1.8.4. For temporal analysis of the full 520 genomes, we used BEAST 2.6.3 with BEAGLE libraries. To perform date randomisation testing of our temporal analysis, we used the TIPDATINGBEAST v1.1-0 package. For analysis of temporal signal and root-to-tip correlations, we used the ggtree v2.5.1 package in R v3.6.0. To subsample and combine BEAST log and tree files, we used logcombiner v2.6.3 (for BEAST2 runs) and logcombiner v1.8.4 (for BEAST1 runs). All runs were compared in Tracer v1.7.1. Final consensus trees were constructed using treeannotator v1.8.4. To explore the proportion of BEAST trees supporting population expansion and decline, we used the scripts population_increase_distribution_BEAST.py and

population_change_support_BEAST.py, both available at https://github.com/chrisruis/tree_scripts commit:2463656e329e3f25ec6dd13c86c64ad163525ae0.

To infer the presence of macrolide resistance alleles, we used the scripts competitive_mapping_Treponema23S--mod.sh, bcf-summarise-specific-sites.py and run_Treponema-23S_competitive-mapping_as_array.sh (available at https://github.com/matbeale/Lihir_Treponema_2020/competitive_mapping_Treponema23S--mod.sh commit:044b29ce29ada81e4f7cb0318301e97e1d5a8d55).

To determine the SNPs between ancestral nodes of contemporary SS14 and Nichols lineages, we used TreeTime v0.7.4 to perform ancestral reconstruction on our maximum likelihood phylogeny. We extracted SNPs from the resulting multiple sequence alignment using snp-sites, functionally annotated variants using SnpEff v4.3 with the most recent NCBI annotation for the NC_021508.1 SS14 reference genome (June 2021), and imported data into R48 v3.6.0 for analysis using vcfR v1.12.0.

To infer pairwise SNPs between core genomes, we used pairsnp v0.1.0 (available at https://github.com/gtonkinhill/pairsnp commit: 0acddba060cc076946dab9969a95ab3c21f110fb). Networks of minimum SNPs were constructed using code in base R v3.6.0. Nucleotide diversity for different multiple sequence alignments was calculated using the EggLib v3.0.0b21 library in python2.7 - custom script available at https://github.com/matbeale/Contemporary_Syphilis_Lineages_2021/scripts. To determine country centroid GPS coordinates, we used the CoordinateCleaner v2.0-17 package in R. We calculated pairwise geographic distances using the geosphere v1.5-10 package in R. Pearson's R Correlations were calculated using the 'cor' function in base R. Maps were generated using ggmap v3.0.0 using the get_stamenmap function. Tanglegrams were plotted using ape, phytools v0.7-47 and ggtree v2.5.1. Phylogenies were plotted using ggtree v2.5.1. All plots made use of ggplot2 v3.3.2, and multi-panel figures were constructed using cowplot v1.1.1.

All R code used for statistical analysis and plotting is available in an Rnotebook at DOI:10.6084/m9.figshare.14376749 and at https://github.com/matbeale/Contemporary_Syphilis_Lineages_2021, along with the raw source data files.

For manuscripts utilizing custom algorithms or software that are central to the research but not yet described in published literature, software must be made available to editors and reviewers. We strongly encourage code deposition in a community repository (e.g. GitHub). See the Nature Portfolio guidelines for submitting code & software for further information.

Data

Policy information about availability of data

All manuscripts must include a data availability statement. This statement should provide the following information, where applicable:

- Accession codes, unique identifiers, or web links for publicly available datasets
- A description of any restrictions on data availability
- For clinical datasets or third party data, please ensure that the statement adheres to our policy

Sequencing reads for all novel genomes have been deposited at the European Nucleotide Archive (https://www.ebi.ac.uk/ena/browser/home) in BioProjects PRJEB28546, PRJEB33181 and PRJNA701499. All accessions, corresponding sample identifiers and related metadata are available in Supplementary Data 1. Map tiles were downloaded from http://maps.stamen.com using the ggmap interface. Publicly available syphilis incidence data is available for England at https://www.gov.uk/government/statistics/sexually-transmitted-infections-stis-annual-data-tables and for British Columbia at http://www.bccdc.ca/health-professionals/data-reports/sti-reports.

All sample metadata and intermediate analysis files are available at DOI:10.6084/m9.figshare.14376749 and https://github.com/matbeale/Contemporary_Syphilis_Lineages_2021. The minimum raw datafiles required to construct the Main and Extended Figure are described in Supplementary Data 4. The finescale maximum likelihood phylogeny and metadata are also available for interactive visualisation at https://microreact.org/project/xt7AuLJorkyBNHVXL2sF8G/1a515b2c.

Field-specific reporting

Please select the one below that is the best fit for your research. If you are not sure, read the appropriate sections before making your selection.

☒ Life sciences ☐ Behavioural & social sciences ☐ Ecological, evolutionary & environmental sciences

For a reference copy of the document with all sections, see nature.com/documents/nr-reporting-summary-flat.pdf

Life sciences study design

All studies must disclose on these points even when the disclosure is negative.

Sample size

Sample size calculations were not performed, and this study was performed using the maximum number of available genomes with sufficient coverage and quality for robust phylogenetic reconstruction. For basic lineage assignment (where only a low number of genomic sites are required to cluster genomes) we required that samples had >25% of genomic sites with >5x (5 independent reads, with at least 2 on each strand) - 726 samples met these criteria. With the exception of the 'Nichols reference genomes', we also excluded samples known to be duplicates (e.g. the same sample or derivative has been sequenced multiple times in the literature or in our dataset) - where this was known to be the case, we included the readset with the highest coverage, or if given a choice between a readset or a published assembly (which would require simulated reads to be generated), we preferentially selected reads over assemblies, unless the reads were of low coverage.

For more detailed phylogenetic reconstruction, we required all genomes had >75% of genomic sites >5x coverage (5 independent reads with >2 reads on each strand) - 528 samples met these criteria. For temporal analysis, although our analysis showed that passage in the rabbit model did not affect the overall phylogeny, extensive passage in the rabbit model was expected to affect coalescent age, since the exact

duration of passage (relative to the original sampling date) was usually unknown - using 1912 for the Nichols derivatives, passaged for decades to an uncertain number of generations was clearly inappropriate. We therefore excluded these genomes from temporal analysis (as well as three further samples lacking accurate collection dates), leaving a final dataset of 520 genomes for temporal analysis.

We also performed temporal analysis on subsets of the data, stratifying samples by sublineage, country and sample year, then using a random sampler in R to create subsampled datasets for analysis - the findings between our full dataset and the subsampled datasets were very similar, with overlapping confidence intervals. Finally, we performed stratified BEAST analyses on important sublineages, creating new datasets based on sublineages for additional analysis using equivalent parameters.

There are a small number of ancient TPA genomes published (e.g. published in Schuenemann/Majander et al), and these might have informed our temporal analyses, but the coverage for all these genomes was very low and did not pass our criteria for assessment.

Data exclusions

We did not attempt to sequence samples with qPCR Ct >32, due to known limitations of the sequence capture methods. We sequenced some samples that did not meet our minimum quality criteria for inclusion, and we also excluded low quality genomes published by others according to the criteria described above.

Replication

All Maximum likelihood phylogenies were assessed using 10,000 UltraFast bootstraps, where a UFboot value >95% indicates strong support - these are indicated on all ML trees. All BEAST trees were shown to converge, and we compared the tree topology and inferred clock rates and dates between the full dataset and subsampled datasets.

Randomization

We performed tip date randomisation tests from our full 520 genome dataset, generating 20 datasets with tip dates randomised for replicate analysis in BEAST - these random datasets showed no temporal signal, supporting the accuracy of our 'true' dataset.

To enhance the reproducibility of our rPinecone clustering, we generated 100 bootstrapped datasets for independent analysis, and used hierarchical clustering to determine the optimal cluster assignments of samples.

For analysis of the relationship between pairwise SNP distances and geographic (and temporal) distance, we performed two-tailed Pearson's correlation tests on the real data, then repeated each analysis with 1000 randomised datasets to provide a baseline for comparison.

Blinding

Sequencing teams were blind to group allocation, country, sample date, etc. The majority of the study was performed using metadata agnostic phylogenetic methods, and metadata were included only after initial analysis. Initial phylogenies were calculated solely on the genomic data. Phylogenetic clustering (rPinecone) was performed on these phylogenies. Subsequent analyses (e.g. temporal) required the inclusion of sample metadata such as collection year, and were therefore not blinded.

Reporting for specific materials, systems and methods

We require information from authors about some types of materials, experimental systems and methods used in many studies. Here, indicate whether each material, system or method listed is relevant to your study. If you are not sure if a list item applies to your research, read the appropriate section before selecting a response.

Materials & experimental systems

n/a	Involved in the study
☒	☐ Antibodies
☒	☐ Eukaryotic cell lines
☒	☐ Palaeontology and archaeology
☒	☐ Animals and other organisms
☐	☒ Human research participants
☒	☐ Clinical data
☒	☐ Dual use research of concern

Methods

n/a	Involved in the study
☒	☐ ChIP-seq
☒	☐ Flow cytometry
☒	☐ MRI-based neuroimaging

Human research participants

Policy information about studies involving human research participants

Population characteristics

Patients with clinically diagnosed syphilis and PCR-positive Treponema pallidum lesions. The full dataset includes publicly available genomes, samples historically isolated through passage in the rabbit model aswell as novel samples sequenced from residual DNA extracted for diagnostic PCRs. Detailed demographic information on patients is not available in most cases (and in other cases, cannot be released due to ethical constraints), but where known, all patients were aged between 16-73, with the exception of five samples historically isolated in rabbits from children/neonates (Haiti B, BAL-2, BAL-6, BAL-8, Grady-1) during the 1950s-1980s and well described in previous literature. For clinically derived samples, we had only very limited information on patient gender, sexual preference, or treatment stage - these metadata were incomplete for all but a small fraction of samples, but from the limited analysis possible, the dataset contained samples collected from both male and female patients, MSM as well as heterosexual men and women, and patients with primary and secondary syphilis. We had no information on prior patient history or treatments used.

Recruitment

With the exception of samples previously isolated in the rabbit model, all samples were obtained through participating laboratories providing residual genomic DNA from previous diagnostic PCRs, or residual samples from previous studies for DNA extraction (Dublin). In some cases, submitting laboratories were National (e.g. UK, Hungary) or Regional (Canada, Sweden, Russia, Zimbabwe) reference laboratories, whilst in other cases we had samples from individual laboratories (Belgium, Ireland, Australia, South Africa, Spain) or historically collected through a network of collaborating labs (USA).

Furthermore, for most countries samples came from a small timespan or a single year - we had detailed longitudinal sampling from USA (1951-2011), Canada (2000-2018) and UK (2012-2018). Moreover, we applied a selection criteria that samples must meet a maximum qPCR Cq of 32 for sequencing due to the known limitations of the sequencing method. Some samples failed to successfully yield a genome, whilst others were of low coverage, and therefore only included in the initial analysis of 726 genomes, but excluded from the subsequent finescale analyses. Our samples were therefore not necessarily representative of each country, but provide a snapshop at a particular point in time of the diversity sampled by that particular laboratory, and it is possible (and indeed likely) that changes occur over time or with greater sampling (this is one of the major points addressed in the manuscript).

Samples were deidentified and not linked to any personal identifiable information. As no patient contact took place, no change to clinical care occurred and the study consists only in the use of residual DNA from samples which were already routinely collected, patient consent was deemed unnecessary during ethical approval.

Ethics oversight

Overall Ethical ethical approval for receipt, handling and sequencing of all clinical samples, as well as for use of UK samples collected as part of public health surveillance and for research was granted by the London School of Hygiene and Tropical Medicine Observational Research Ethics Committee (REF#16014) and the National Health Service (UK) Health Research Authority and Health and Care Research Wales (UK; 19/HRA/0112). Samples were deidentified and not linked to any personal identifiable information. As no patient contact took place, no change to clinical care occurred and the study consists only in the use of residual DNA from samples which were already routinely collected, patient consent was deemed unnecessary during ethical approval. Ethical approval for sequencing the samples from Belgium was covered by a provision of the Institutional Review Board of the Institute of Tropical Medicine that allows the further characterization of residual patient samples without additional Ethical Committee clearance. In addition, at the Institute for Tropical Medicine outpatient clinic the patients are informed that their remnant samples may be used; if they do not consent they have a form to complete (opt out). Samples from Hungary were collected and preserved as part of the routine diagnostics (standard care), and stored at laboratories which have approval for preservation of such and other clinical samples, and no patient identification information was available - accordingly, these samples do not need a separate ethical approval for use in an anonymised manner. Samples from Russia were collected as part of a previous study that involved molecular epidemiology, and this had ethical approval from The State Research Center of Dermatology, Venereology and Cosmetology of The Russian Ministry of Health (SRCDVC), Moscow, Russia. Samples from South Africa were collected as part of a study on the impact of episodic acyclovir therapy on ulcer duration & HIV shedding from genital ulcers in men, and ethical approval was granted by the Human Research Ethics Committee of the University of the Witwatersrand in South Africa (Clearance Certificate Nos: M040548 and M10201). All participants gave permission to store samples for future testing for infectious diseases. Zimbabwe samples were collected as part of the Zimbabwe STI Etiology Study, which had a provision for specimen storage and future studies and the consent form had a specific opt-in/opt-out addendum for specimen storage and future studies. It also specifically asked for consent to have specimens shipped to NICD in South Africa. The protocol and consent forms were approved by the Research Council and Medical Research Council of Zimbabwe. Samples from Canada (British Columbia and Alberta) were collected as part of public health surveillance, were deidentified before transfer between labs, and were deemed exempt from requiring additional ethical approval. Samples from Australia were covered by HREC approval, and that this approval included a waiver to obtain individual informed consent that was consistent with the requirements outlined in the Australian NHMRC National Statement. For samples from Spain, all the patients enrolled in provided written consent for collection of an additional ulcer swab and/or whole blood specimen to perform the TPA molecular studies. Institutional Review Board approval PR(AG)297/2014 was obtained from the Ethics Committee of Vall d'Hebron Research Institute. An amendment was also approved to allow WGS. For samples from Ireland, the study was approved by the ethics board of St James's Hospital and Tallaght Hospital, and this included approval for molecular analyses.

Note that full information on the approval of the study protocol must also be provided in the manuscript.
